# Combining lifestyle risks to disentangle brain structure and functional connectivity differences in older adults

**DOI:** 10.1038/s41467-019-08500-x

**Published:** 2019-02-06

**Authors:** Nora Bittner, Christiane Jockwitz, Thomas W. Mühleisen, Felix Hoffstaedter, Simon B. Eickhoff, Susanne Moebus, Ute J. Bayen, Sven Cichon, Karl Zilles, Katrin Amunts, Svenja Caspers

**Affiliations:** 1 Institute of Anatomy I, Medical Faculty, Heinrich-Heine-University Duesseldorf, Duesseldorf, 40225 Germany; 2 Institute of Neuroscience and Medicine (INM-1/INM-7), Research Centre Juelich, Juelich, 52425 Germany; 3 Department of Psychiatry, Psychotherapy and Psychosomatics, RWTH Aachen University, Aachen, 52074 Germany; 4 JARA-BRAIN, Juelich-Aachen Research Alliance, Juelich, 52425 Germany; 5 C. and O. Vogt Institute for Brain Research, Heinrich-Heine-University Duesseldorf, Duesseldorf, 40225 Germany; 6 Human Genomics Research Group, Department of Biomedicine, University of Basel, Basel, 4031 Switzerland; 7 Institute of Systems Neuroscience, Heinrich-Heine-University Duesseldorf, Duesseldorf, 40225 Germany; 8 Institute of Medical Informatics, Biometry and Epidemiology, University of Duisburg-Essen, Essen, 45122 Germany; 9 Mathematical and Cognitive Psychology, Institute of Experimental Psychology, Heinrich-Heine-University Duesseldorf, Duesseldorf, 40225 Germany; 10 Institute of Medical Genetics and Pathology, University Hospital Basel, Basel, 4031 Switzerland

## Abstract

Lifestyle contributes to inter-individual variability in brain aging, but previous studies focused on the effects of single lifestyle variables. Here, we studied the combined and individual contributions of four lifestyle variables - alcohol consumption, smoking, physical activity, and social integration - to brain structure and functional connectivity in a population-based cohort of 549 older adults. A combined lifestyle risk score was associated with decreased gyrification in left premotor and right prefrontal cortex, and higher functional connectivity to sensorimotor and prefrontal cortex. While structural differences were driven by alcohol consumption, physical activity, and social integration, higher functional connectivity was driven by smoking. Results suggest that combining differentially contributing lifestyle variables may be more than the sum of its parts. Associations generally were neither altered by adjustment for genetic risk, nor by depressive symptomatology or education, underlining the relevance of daily habits for brain health.

## Abstract (web summary)

Lifestyle factors such as smoking and exercise contribute to the health of the brain during aging, but previous studies have focused on the effects of single lifestyle variables. Here, the authors examine the combined and individual effects of four lifestyle variables on brain structure and function.

## Introduction

Structural decline and functional reorganization are major hallmarks of brain aging. Both show high inter-individual variability, especially in later decades of life. Here, lifestyle habits came into focus possibly influencing this variability^1^. While some lifestyle habits may pose serious risks to brain health, others may be protective. Physically more active older adults show less volume loss^2,3^, and better performance in cognitive tasks^4^ along with higher task-related activity in attentional networks^5^. Likewise, stronger social integration of older adults is associated with reduced cognitive decline^1^, reduced risk of dementia^1^ and Alzheimer’s disease^6^, and higher regional and overall gray matter (GM) volumes^7,8^. Both lifestyle habits therefore seem to promote cognitive or neural reserve capacity^9,10^, that is, the ability to tolerate age or disease load without functional impairments. In contrast, other lifestyle habits may pose serious risks to healthy brain aging. Studies reported associations between heavy smoking and cortical thinning^11^ or lower GM density^12^. In addition, smokers, compared to non-smokers, showed reduced resting-state functional connectivity (RSFC)^13^, as a correlate of generally altered functional brain architecture^14^, between the insula and prefrontal cortex. Similarly, chronic alcohol dependence can lead to severe neurological diseases, for example, Korsakoff syndrome^15^. GM loss, however, has also been reported in healthy older adults with non-dependent alcohol consumption^16^. In alcohol-dependent patients^17^, regional GM loss was found particularly in the frontal cortex, whereas white matter (WM) loss is more pronounced in corpus callosum and cerebellum^17^. Further, RSFC as well as performance in a simple motor task and associated brain activation were found to be decreased in alcohol-dependent patients^18^. Hence, alcohol consumption and smoking may both be variables accelerating brain aging and reducing brain reserve.

Previous studies mainly investigated effects of single lifestyle variables in isolation. However, individuals rather show a combination of lifestyle habits that could all possibly influence brain reserve, for example, being a smoker (risk) and socially and physically active person (protective) versus being a smoker and an inactive person. Yet, studies examining combinations of lifestyle variables are rare. One study found pronouncedly different RSFC, particularly when participants who both smoked and consumed alcohol were compared to participants with only one of these risk variables^19^. This underlines the notion that individual lifestyle variables may have intermingling effects on the aging brain. It is therefore essential to examine combinations of lifestyle habits to understand the high inter-individual variability in the reserve capacity to tolerate age-related differences.

**Fig. 1:**
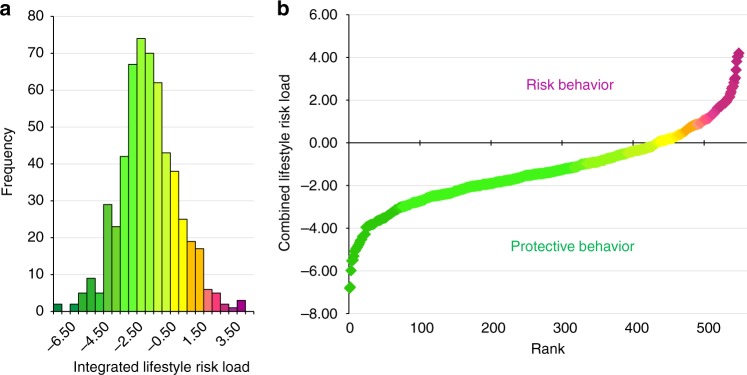
Combined lifestyle risk measured with the developed combined lifestyle risk score. Lifestyle related data, that is, alcohol consumption (g/week), smoking (pack years), social integration (social integration index^23^), and physical activity (metabolic equivalent per week^22^) were *z*-transformed to obtain uniform measure units. *Z*-transformed data on the protective variables physical activity and social integration were first multiplied with (−1) and then summed up with *z*-transformed data on the risk variables of alcohol consumption and lifetime smoking. This resulted in an individual score of combined lifestyle risk. **a** The combined lifestyle risk score shows a nearly normal distribution. **b** Rank-sorted data of the combined lifestyle risk score: The participant with the lowest score gets rank 1 and the highest rank 549. Green colors indicate more protective lifestyle behavior and magenta colors indicate more risky lifestyle behavior

Consequently, we developed a combined lifestyle risk score to investigate the relation between lifestyle risk as a combined concept and brain aging. Based on the literature described above, physical activity^2–5^ and social integration^1,6–8^ were classified as protective variables, and alcohol consumption^15–18^ and smoking^11–13^ as risk variables. Data on all four lifestyle variables were assessed via self-report in 549 older participants (248 female) aged 55 to 85 years from the population-based 1000BRAINS cohort study^20^. Physical activity was examined as metabolic equivalent^21^, alcohol consumption in grams of consumed alcohol per week, smoking as pack years, and social integration as the social integration index^22^. Similar to the concept of genetic risk scores, we combined these four lifestyle variables into one risk score indicating combined lifestyle risk (Fig. 1). Negative values indicated a rather protective lifestyle (e.g., high levels of physical activity and social integration, plus low alcohol consumption and no smoking), and positive values a combination of more risky behaviors. Further details can be found in the Methods section.

To investigate the relation between lifestyle and brain structure and function, we tested not only this combined risk score but also investigated the contributions of each lifestyle variable. To this end, we successively excluded each lifestyle variable from the combined lifestyle risk score resulting in eight risk score models with different combinations of single lifestyle variables (see Methods and Supplementary Table 1): Four risk score models included three single variables and another four included two single variables, where always one protective and one risk variable were combined to examine whether they canceled each other out^19^. We used this epidemiologically motivated comprehensive operationalization of lifestyle in three consecutive analyses to understand its relation to reorganization during brain aging: Analysis of (1) brain structure regarding (a) gyrification and (b) subcortical volumes, (2) RSFC, and (3) both analyses while additionally accounting for genetic risk.

Recent studies identified the local gyrification index (LGI)^23^, the degree of cortical folding, as a sensitive surface-based measure^24,25^ for studying age-related differences in local brain structure. Differences in arealization and gyrification seem to be more closely related to age than differences in cortical thickness (CT)^24^. Higher gyrification is supposed to promote functional development and brain connectivity more efficiently than increasing cortical GM^24^. In turn, lower gyrification would imply lower brain reserve capacity. We therefore hypothesized (1a) that higher combined lifestyle risk would be associated with lower local gyrification. We tested this hypothesis in FreeSurfer^26,27^ based on T1-weighted structural magnetic resonance (MR) images. To complement this surface-based approach, we tested for an association between combined lifestyle risk and volume of subcortical structures (1b), as well as (1c) CT as a second surface parameter^28^ adding the dimension of cortical GM volume in supplementary analyses. Our second analysis examined whether the identified lifestyle-related variations in brain structure would be accompanied by variations in functional connectivity (2). Here, we tested for decreases in RSFC, as well as RSFC increases that have been repeatedly reported in pathological conditions^14^. RSFC increases that already occur during rest may reflect higher base levels that leave no room for additional increases in brain activity during active tasks to boost performance^9,28^. Therefore, higher base levels supposedly reflect lower cognitive reserve^29,30^ and would be expected in individuals with higher combined lifestyle risk. We hence hypothesized that those regions that showed lifestyle-related variations in cortical folding would also show variations in RSFC. Finally, evidence for influences of genetic susceptibility on variability in behavior^31–34^ and brain organization^35^ has recently been provided. To account for individual genetic susceptibility (3), we constructed a polygenetic risk score (PRS) from genome-wide association studies (GWAS, see Methods) of smoking and alcohol consumption.

The current study shows that combined lifestyle risk is associated with decreased gyrification in left premotor and right prefrontal cortex, and related higher functional connectivity with sensorimotor and prefrontal cortex. While decreased gyrification was driven by alcohol consumption, physical activity, and social integration, higher functional connectivity was driven by smoking. Neither genetic influences nor a set of non-lifestyle variables (depressive symptomatology, Beck Depression Inventory-II (BDI-II)^36^ and education level as measured with the international standard classification of education (ISCED)^37^) modulated the relation between combined lifestyle risk and the brain phenotypes. Daily lifestyle habits and their interplay therefore seem to influence structural and functional brain health in older adults, beyond other influencing factors.

## Results

### Sample characteristics

Mean age of the final study sample (*n* = 549) was 67.4 years (SE = 0.28, Table 1). Mean combined lifestyle risk (summed-up *z*-scores) was −1.30 (SE = 0.07) and its distribution did not significantly deviate from normality (Kolmogorov–Smirnov test: *p* = 0.226, Fig. 1). Data on all four single lifestyle variables (Table 1) were considerably skewed due to a substantial number of participants not engaging in the specific lifestyle behavior, for example, 216 (136 female) participants did not consume alcohol. Among these 216 participants, 16 never consumed alcohol and no participant reported abstinence due to former alcohol dependence. Similarly, 255 (139 female) participants never smoked. We used subgroups of the study sample for the analysis of RSFC (*n* = 501) and for the PRS analysis (*n* = 488), since RSFC and PRS were not available for all participants.

**Table 1:** Descriptive statistics of lifestyle variables

Lifestyle variable	Mean	SE	Min	Max
Alcohol consumption (in g/week)	70.00	4.41	0.00	952.77
Smoking (in pack years)	12.77	0.80	0.00	120.00
Social integration (as social integration index)	12.72	0.25	2.00	43.00
Physical activity (metabolic equivalent per week)	40.75	1.71	0.00	257.75
Combined risk score (combined *z*-scores)	−1.30	0.07	−6.81	4.20

### Combined lifestyle risk and gyrification

**Fig. 2:**
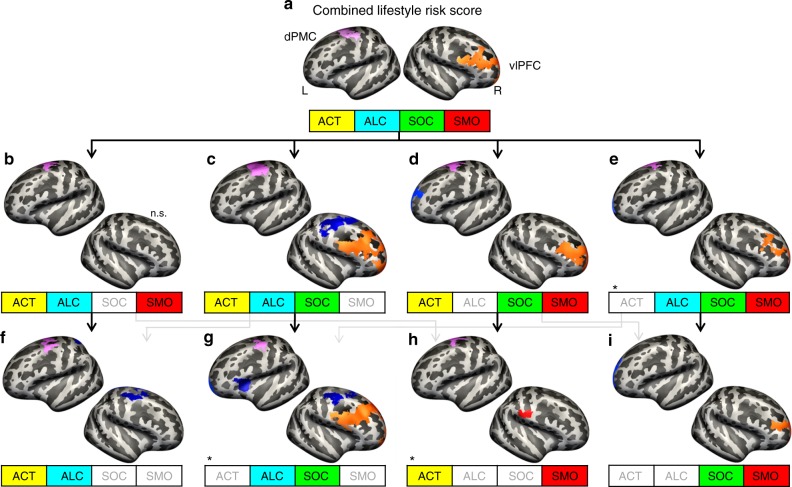
Differences in cortical folding associated with lifestyle risk in different risk score models. **a**–**i **Small boxes represent different risk score models derived from the stepwise exclusion approach with excluded single lifestyle variables grayed out. Associations between cortical folding and the combined lifestyle risk score, or the different risk score models, respectively, are depicted on the inflated surfaces of the fsaverage brain. Arrows indicate the work flow of the stepwise exclusion. Negative associations are represented in blue, and positive associations in red. The recurrent negative associations between higher lifestyle risk and reduced cortical folding in left dorsal premotor cortex (dPMC) and right ventro-lateral prefrontal cortex (vlPFC) are colored in pink and orange, respectively. All results are corrected for age and gender and corrected for multiple comparisons using Monte Carlo Z simulations with *α* = 0.05. Some of the associated regions in specific risk score models marked with an asterisk did not pass an additional correction for multiple comparisons at *α* = 0.05/13 = 0.0033. For detailed information see Supplementary Table 2. n.s. = not significant, ACT = physical activity, ALC = alcohol consumption, SOC = social integration, SMO = pack years of smoking, L = left hemisphere, R = right hemisphere

All reported analyses were statistically corrected for age and gender as covariates and corrected for multiple comparisons using Monte Carlo simulations at *α* = 0.05 with a cluster-wise *p* value (cwp) < 0.01 (two-sided test, for uncorrected results, see Supplementary Fig. 1). A higher combined lifestyle risk score was associated with lower local gyrification in two distinct cortical areas, namely left dorsal premotor cortex (dPMC, cwp *=* 0.0001, Fig. 2a) and ventro-lateral prefrontal cortex (vlPFC, cwp = 0.0001, Fig. 2a) extending from the frontal pole to middle frontal gyrus and to posterior inferior frontal gyrus and sulcus.

Stepwise exclusion (Fig. 2) of one or more lifestyle variables from the combined score and examining the associated differences in gyrification revealed a consistent pattern: (i) Higher lifestyle risk in all risk score models including alcohol consumption and/or physical activity (Fig. 2a–h) was associated with decreased cortical folding in left dPMC. This association disappeared, when excluding both alcohol consumption and physical activity (Fig. 2i). This hinted at alcohol consumption and physical activity being the driving behaviors behind this association. (ii) Lower lifestyle risk in all risk score models that included social integration (Fig. 2a, c, d, e, g, i) was consistently associated with decreased cortical folding in right vlPFC. Since this association vanished when social integration was excluded (Fig. 2b, f, h), this hinted at social integration as the main driving behavior for this association. To further test this association, we extracted cortical folding values of the vlPFC region and used them as dependent variable in a post-hoc multiple linear regression including all four single lifestyle variables, age, and gender as regressors. With *β* = 0.11, social integration was the strongest predictor of cortical folding in vlPFC (*F* test, *F*(1,542) = 3.95, *p* = 0.0007; for details, see Supplementary Fig. 17).

We did not find any significant association between single lifestyle variables and cortical folding using permutation-based inference. Uncorrected results are shown in Supplementary Fig. 2. Analyses including additional covariates and measure (CT) can be found in Supplementary Figs. 11–16.

### GM volume of subcortical areas

The strongest association was found between enhanced social integration alone and greater GM volume of left hippocampus (partial Spearman’s *ρ* = 0.15, *p* = 0.0017). This was the only correlation surviving a post-hoc Bonferroni correction using a threshold of *α*_corr_ = 0.05/20 = 0.0025 with 20 subcortical structures tested (Table 2).

**Table 2:** Correlations between lifestyle risk score models and subcortical structures

Risk score model	Hemisphere	Subcortical structure	*r* (correlation)	*p* value
Combined risk score, ACT, ALC, SMO, SOC	Right	Amygdala	−0.086	0.044
		Nc. accumbens	−0.100	0.019
ACT, ALC, SMO	Left	Thalamus	0.089	0.037
ACT, ALC, SOC	Right	Amygdala	−0.087	0.042
ACT, SOC, SMO	Left	Hippocampus	−0.089	0.037
		Putamen	−0.092	0.030
		Globus pallidus	−0.089	0.037
	Right	Nc. accumbens	−0.084	0.049
		Hippocampus	−0.091	0.033
		Putamen	−0.089	0.036
ALC, SOC, SMO	Left	Hippocampus	−0.091	0.033
	Right	Nc accumbens	−0.129	0.003
		Putamen	−0.088	0.040
ACT, ALC	Bilateral	Mid anterior cingulum	−0.105	0.013
ALC, SOC	Left	Hippocampus	−0.105	0.014
		Amygdala	−0.105	0.014
SOC, SMO	Left	Hippocampus	−0.107	0.012
		Globus pallidus	−0.096	0.024
	Right	Putamen	−0.098	0.022
		Nc. accumbens	−0.108	0.012
ACT as single variable	Bilateral	Anterior cingulum	0.107	0.012
SMO as single variable	Right	Nc. accumbens	−0.101	0.018
SOC as single variable	Left	Hippocampus^a^	0.134^a^	0.002^a^
		Amygdala	0.088	0.039
	Right	Hippocampus	0.101	0.018
		Nc. caudatus	0.12	0.005

Table shows significant partial correlations between lifestyle risk score models and subcortical structures, corrected for age, gender, and total intracranial volume, *n* = 549

Nc: nucleus, *r*: Spearman's correlation coefficient

^a^The partial correlation between social integration and the left hippocampus was the only one being significant at an *α*-level corrected for multiple comparisons *α*_corr_ = 0.05/20 = 0.0025. The complete correlation matrix can be found in Supplementary Data 1

### Combined lifestyle risk and RSFC

We used regions showing variations in cortical folding in relation to the combined lifestyle risk score as seed regions (dPMC and vlPFC) to test for variations in RSFC to all other GM voxels. All reported associations were significant at *α* = 0.05 (cluster level corrected, cluster-forming threshold *α* < 0.001, two-sided) and corrected for age and gender. Decreased RSFC between the seeds and other regions showed no systematic differences (see Supplementary Figs. 3, 4).

Regarding the seed region of left dPMC, higher combined lifestyle risk scores were associated with increased RSFC between left dPMC seed and bilateral primary motor, left somatosensory, left entorhinal, and left higher visual cortex.

**Fig. 3:**
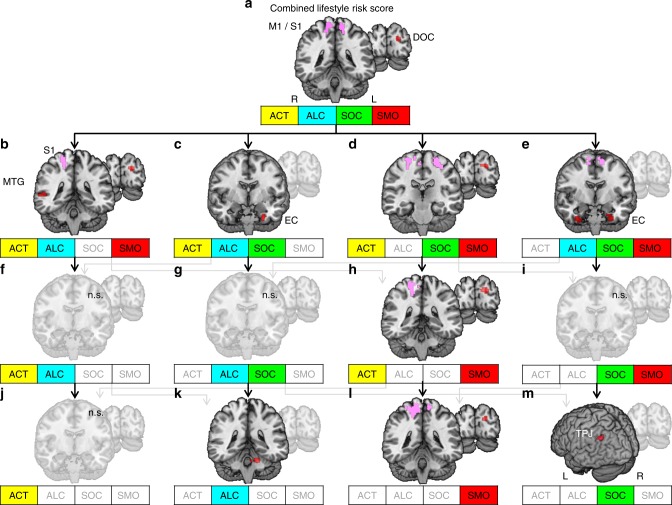
Lifestyle risk-associated increases in resting-state functional connectivity (RSFC) to the seed in the left dorsal premotor cortex (dPMC). **a**–**m** Abbreviations in the small boxes refer to the same variables as in Fig. 2. Coronar sections show brain regions exhibiting increases in RSFC depicted in red. The recurrent pattern of higher RSFC between the dPMC and the somato-motor cortex is highlighted in pink. Arrows indicate the work flow of the stepwise exclusion. Transparent sections represent not significant (n.s.) results. This analysis included a subsample of the study population (*n* = 501). Results were significant at *α* = 0.05 (cluster level corrected, cluster-forming threshold *α* = 0.001). S1 = primary somatosensory cortex, MTG = middle temporal gyrus, M1 = primary motor cortex, DOC = dorsal occipital cortex, EC = entorhinal cortex, TPJ = temporo-parietal junction, L = left hemisphere, R = right hemisphere

Again, we applied our stepwise exclusion approach to test for individual contributions of single lifestyle variables. Excluding social integration, alcohol consumption, or physical activity from the risk score left the overall association between left dPMC and reported regions unaltered (Fig. 3b, d, e, h, l). Contrarily, when excluding smoking from the risk score models, increases in RSFC were no longer found (Fig. 3c, f, g). Further, smoking as a single variable revealed higher RSFC between left dPMC and bilateral primary motor and somatosensory cortex, left superior frontal gyrus, left dorsal and right lateral occipital cortex, that is, clusters that were similarly found in the respective risk score models that included smoking (compare Fig. 3a, b, d, e, h with Fig. 3l). Thus, RSFC was only increased when smoking was considered, either incorporated in a risk score model or as single variable. Besides this general pattern, we found an additional systematic change in RSFC of left dPMC (Supplementary Table 3): All risk score models that included both physical activity and smoking, or smoking as a single variable, were significantly related to higher RSFC between left dPMC and dorsal visual cortex (Fig. 3a, c, d, h, l).

For single lifestyle variables, we found additional associations (Supplementary Table 3): Higher RSFC between dPMC and (i) left rostral superior frontal gyrus (for smoking), (ii) left temporo-parietal junction (for social integration, Fig. 3m), and (iii) left cerebellum (for alcohol consumption, Fig. 3k).

**Fig. 4:**
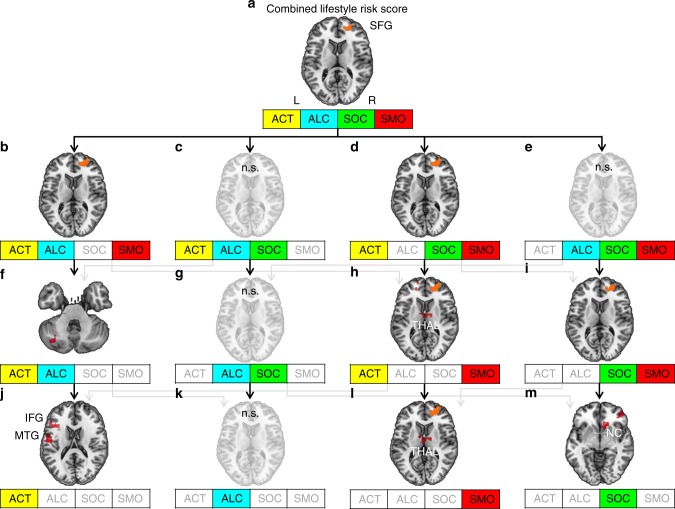
Lifestyle risk-associated increases in resting-state functional connectivity (RSFC) to the seed in right ventro-lateral prefrontal cortex (vlPFC). **a**–**m** Transversal sections show brain regions exhibiting increases in RSFC depicted in red. The recurrent pattern of higher RSFC between right vlPFC and superior frontal gyrus is outlined in orange. Arrows indicate the work flow of the stepwise exclusion. This analysis included a subsample of the study population (*n* = 501). For other conventions, see Fig. 2. Results were significant at *α* = 0.05 (cluster level corrected, cluster-forming threshold *α* = 0.001). SFG = superior frontal gyrus, MTG = middle temporal gyrus, THAL = thalamus, NC = caudate nucleus, n.s. = not significant, L = left hemisphere, R = right hemisphere

Now analyzing RSFC of the vlPFC, this seed showed increased RSFC to right anterior superior frontal gyrus (SFG, Fig. 4a, Supplementary Table 4) in relation to higher combined lifestyle risk.

Again, we applied our stepwise exclusion approach for the seed in right vlPFC. The cluster within right SFG was repeatedly found in all risk score models containing smoking and not found when smoking was excluded (Fig. 4c, f, g). Additionally, this cluster showed higher RSFC in relation to smoking as a single variable (Fig. 4l), as well as clusters in its left homolog and thalamus. Results remained unchanged when removing any other lifestyle variable (Fig. 4b–i).

Two other single variables were associated with increased RSFC to the right vlPFC seed, that is, to right caudate nucleus, and right rostral inferior frontal gyrus (social integration, Fig. 4m); and to left inferior frontal gyrus triangular part, left post-central gyrus, and right middle temporal gyrus (physical activity, Fig. 4j).

### Additional adjustment for polygenic risk

**Fig. 5:**
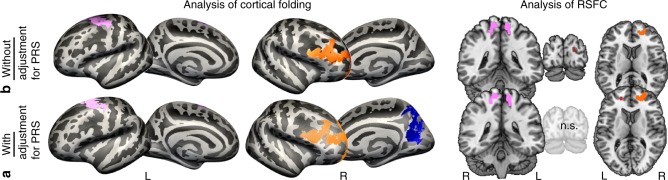
Comparison of analyses without adjustment for polygenetic risk scores (GRS) and with adjustment for polygenetic risk score (PRS). **a** The lower row shows the results of the analyses of cortical folding and resting-state functional connectivity (RSFC) with adjustment for PRS. **b** The upper row shows the results of the analyses of cortical folding and RSFC with adjustment for PRS. Whereas the general pattern of the results did not change after adjustment for PRS, an additional decrease in cortical folding in right precuneus was observed. n.s. = not significant, L = left hemisphere, R = right hemisphere

Entering the polygenic risk score (PRS, composed of genetic risk for alcohol consumption and smoking, see Table 3 in Methods) as a covariate did not change the general pattern of results, but revealed an additional effect in right precuneus as can be seen in Fig. 5 (see Supplementary Fig. 5 and Supplementary Table 7). Results of the RSFC analyses did not change either when adjusting for PRS (Supplementary Figs. 6 and 7, Supplementary Tables 8 and 9).

**Table 3:** Selected SNPs from GWAS of smoking and alcohol consumption

Trait	Study	SNP	Effect size	*p* overall	Min	Maj	Effect	Gene	CHR
SMO	Liu et al.^33^	rs1051730	−0.08	1.71E^−66^	A	G	G	*CHRNA5/3*	4
		rs6495308	0.07	5.82E^−44^	C	T	T	*CHRNA3*	4
	Thorgeirsson et al.^32^	rs13280604	0.31	1.3E^−8^	G	A	A	*CHRNB3*	
		rs4105144	0.39	2.2E^−12^	T	C	C	*CYP2A6*	8
		rs7937	0.24	2.4E^−09^	C	T	T	*RAB4B*	10
		rs7260329	0.20	5.5E^−06^	A	G	G	*CYP2B6*	10
	Tobacco and Genetics Consortium^33^	rs1329650	−0.37	5.67E^−10^	T	G	G	*LOC100188947*	15
		rs1028936	−0.45	1.29E^−09^	C	A	A	*LOC100188947*	15
		rs3733829	0.33	1.04E^−08^	G	A	G	*EGLN2*	15
ALC	Clarke et al.^35^	rs1260326	−0.03	1.34E^−21^	T	C	T	*GCKR*	2
		rs9841829	0.02	3.36E^−10^	G	T	G	*CADM2*	3
		rs11940694	−0.03	8.4E^−19^	A	G	A	*KLB*	4
		rs145452708	−0.03	1.15E^−30^	C	G	C	*ADH1B/c*	4q23
		rs29001570	−0.03	9.58E^−19^	C	T	C	*ADH5*	4q23
		rs35081954	0.02	2.14E^−10^	CTG	C	CTG	*ADH1c*	4q23
		rs193099203	−0.03	3.79E^−25^	T	C	T	Intergenic	4

SMO: smoking (cigarettes per day), SNP: single-nucleotide polymorphism, GWAS: genome-wide association studies, ALC: alcohol consumption (g/day), Min: minor frequency allele, Maj: major frequency allele, CHR: chromosome

## Discussion

This study examined the relation between combined lifestyle risk and cortical folding and RSFC of the aged brain. Four core findings emerged: First, the combined lifestyle risk score showed significant systematic associations to regional cortical folding, whereas single lifestyle variables did not. Second, physical activity and alcohol consumption were the main variables contributing to reduced cortical folding in left dPMC, and third, social integration was the main variable contributing to higher cortical folding in right vlPFC as revealed by the stepwise exclusion approach. These patterns were highly stable, observed in the main as well as in all additional and sensitivity analyses, as often used in epidemiological research (see Supplementary Methods). Fourth, systematic alterations in RSFC were mainly influenced by smoking. Finally, adjustment for genetic risk for smoking and alcohol consumption did not alter the general pattern of results.

Our first finding that higher combined lifestyle risk scores showed significant systematic associations to lower regional cortical folding, whereas single lifestyle variables did not, may be explained by additivity: Many different influencing factors contribute to variations in older adults' brains^38^, with each variable explaining only a small amount of variance. Hence, single effects of each lifestyle variable on cortical folding might be too small to reach significance. By combining different lifestyle variables, their effects on the aged brain might either add up or integrate—becoming strong enough to reach significance. Another reason for finding effects for the combined, but not for single lifestyle variables might be that the combined lifestyle risk score reduces noise by integrating underlying information from different single lifestyle variables. Reasons explaining why we did not replicate previously reported results on single lifestyle variables might be manifold, for example, the population-based and therefore heterogeneous nature of our sample in contrast to intervention studies often having very homogenous samples^8^. Further, we did not investigate lifestyle behavior in patients^6,17^, but in healthy older adults, where pathological differences may be subtler.

Our second core finding that higher combined lifestyle risk was associated with lower cortical folding in left dPMC is significant, but only when alcohol consumption and/or physical activity were included into the risk score models. Importantly, neither physical activity nor alcohol consumption as single variables were significantly related to cortical folding. Thus, both seem to drive the association between lifestyle risk and cortical folding only in combination with other lifestyle risk variables. Interestingly, we found the same association between physical activity and alcohol consumption and decreases in brain structure also with respect to decreased CT (Supplementary Fig. 14). Physical activity has repeatedly been linked to better cognitive performance^4^ and reduced age-related GM loss^3^. It has been discussed that physical activity promotes increase or preservation of brain structure^3,4^, analogous to “activity”-induced or “training”-induced structural adaptations of neuronal tissue described in animals^39^ and humans^40^. This increase in GM was replicated in older adults^41^, suggesting that structural adaptations are even possible in later life. A similar relation has also been described in the “use it or lose it” hypothesis^42^ regarding cognitive or motor abilities. According to this hypothesis, the more particular abilities are used during daily life, the better they are preserved in the course of aging. Consequently, often engaged brain regions show structural adaptation^40,43^ and are better preserved^42^ also in late life^44^, which contradict the theory of often used brain regions suffering from faster decline^42^. Consistent with the current study, structural correlates of physical activity have been found in PMC^44,45^. The dPMC is involved in a variety of processes needed for daily physical activities and sports, such as movement planning, control, and learning (e.g., dancing), sensorimotor transformations, and action selection^46,47^. Possibly, older adults more engaged in sports and physical activity in daily life recruit the resources of PMC more often. This could lead to better preservation of brain tissue in the very same area, such that an age-related decrease^2^ would be less pronounced in physically more engaged older adults — similar to activity-induced structural adaptations.

**Fig. 6:**
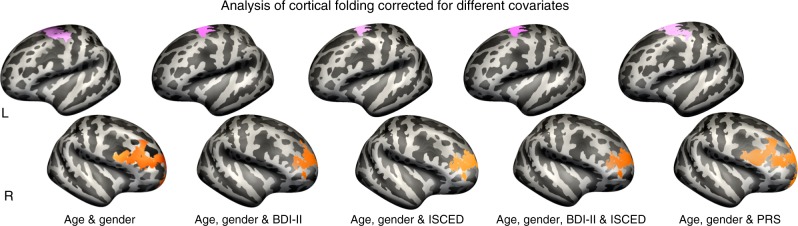
Analyses of cortical folding with additional adjustment for a set of non-lifestyle covariates. The illustrations show the associations between the combined lifestyle risk score and cortical folding when correcting for a set of non-lifestyle covariates. The additional adjustments did not change the general associations between the combined lifestyle risk and decreased cortical folding within the left dorsal premotor cortex (dPMC) (pink) and the right ventro-lateral prefrontal cortex (vlPFC) (orange). BDI-II = depressive symptomatology, as measured with Beck’s depression inventory-II^36^, ISCED = educational level as measured with international classification of education^37^, PRS = polygenic risk score

The other variable contributing to cortical folding in dPMC in the healthy older adults of the present study was alcohol consumption. Chronic alcoholism has repeatedly been related to neurodegeneration, for example, of frontal GM^16^, cerebellar Purkinje cells^48^, and motor performance impairment^18^. However, a direct association between alcohol abuse and degeneration of PMC was not shown previously. A recent study, though, did find decreases in RSFC of motor networks in smoking alcohol consumers^19^. In the present study, older adults with stronger drinking habits showed lower cortical folding in dPMC and exhibited increased RSFC between dPMC and cerebellum (Fig. 3l). In our study, we found increased alcohol-related RSFC in a fronto-cerebellar network already during rest. As previously described, increases in RSFC may hint at lower cognitive reserve^9^ capacity, leaving no room for compensatory increases in brain activity during task performance (compensation-related utilization of neural circuit’s hypothesis^29^, CRUNCH). Altogether, this hints at impairment of (pre-) motor system organization in older adults with stronger drinking habits. This may also explain why alcohol consumers, compared to controls, need to recruit additional brain regions to perform simple motor tasks^18^. Future studies on the interplay between alcohol consumption and motor networks in older adults may possibly provide new insights into how “normal” alcohol consumption influences the aged brain. Interestingly, the association between physical activity, alcohol consumption, and left dPMC was not as stable as the association between social integration and right vlPFC when additionally correcting for depressive symptomatology and education (Supplementary Figs. 11–13), even though the general association between lifestyle risk and cortical folding did not change (Fig. 6). Hence, depressive symptomatology and education may additionally contribute to this more complex association.

The third core finding was the association between lower combined lifestyle risk and reduced decreases in cortical folding in right vlPFC, mainly driven by enhanced social integration. A post-hoc multiple linear regression further confirmed social integration as the strongest explanatory variable of cortical folding within vlPFC (see Supplementary Methods). Consistent with this finding, larger brain volume was found in more socially engaged older adults^7^ and after a social activity intervention for older adults^8^. Several mechanisms might drive this association: Social integration may attenuate age-related neuronal loss^7^, leading to greater brain volume, which is generally considered a factor contributing to brain reserve^9,10^. Animal studies on enriched environment including possibilities for physical and social activities, have indeed shown that socially integrated animals exhibited greater neurogenesis and better spatial learning than isolated animals^49^. Similarly, experimentally enlarged social network size in macaques increased GM volume within temporal and prefrontal cortex and concurrently lead to increased functional connectivity^50^. Social integration may also contribute to brain reserve more indirectly by providing an enriched and cognitively stimulating environment, for example, via cognitive stimulation provided by social activities, better health support, or less depression due to emotional support^1^.

In the current study, a negative correlation between combined lifestyle risk and gyrification was found in right vlPFC, which comprises regions associated with executive networks^51^, attentional control, decision making, and prediction of future outcomes^52^. All of these abilities play a crucial role in successful participation in social situations, for example, requiring to update information on the dialog partner and the group^53^. Furthermore, social interaction also demands inhibition of inadequate behavior, which in turn is based on goal-directed thinking^52^ and prediction of future outcomes to establish long-term, rather than short-term relationships. Similarly, social interactions require emotional self-regulation through top-down control, a process that also has repeatedly been linked to (particularly right) vlPFC^54^. These social activity related abilities pose a high cognitive load and require executive and integration skills. Since these highly complex abilities are precisely subserved by vlPFC, this may explain why the current study found social integration associated with brain structure of the vlPFC, rather than brain structures more typically associated with the social brain. Similar to the activity-induced structural adaptation of brain regions to skill learning and (exercise) training, particularly those brain regions subserving high cognitive, integrative, and emotional load needed for social interactions, may suffer less from age-related decline due to their reoccurring recruitment. This could be referred to as a “use it—or lose it” hypothesis in aging^41^, and is of special interest since the PFC is particularly vulnerable to aging^55^.

Such activity-induced structural adaptation may also explain the correlation between enhanced social integration and reduced decrease in GM volume in left hippocampus. Socially enriched environmental conditions have been linked to hippocampal neurogenesis in animals^49^. Additionally, the hippocampus is involved in memory and flexible provision of knowledge in complex human social situations^52^. Thus, stimulating environments and memory demands of social activity may slow down its age-related decline. These findings are complemented by a positive correlation between social integration and increased RSFC between dPMC and left temporo-parietal junction (Fig. 3m), a region involved in social cognition and theory of mind^56^.

The general pattern of increases in RSFC associated with higher lifestyle risk, though, was mainly driven by smoking—as revealed by the stepwise exclusion approach. Similar to the increases in RSFC associated with alcohol consumption, the current study observed smoking-related increases in RSFC already during rest. As a result of these higher base levels, older adults who smoke may reach their limits faster during an active task possibly performing worse than non-smokers^9,29^, as their neural reserve might be exhausted. Therefore, smoking and the here observed associated increase in RSFC may reflect less neural reserve capacity and less potential for compensation during active tasks. Since such higher base levels are one major hallmark in brain aging^29^, they may even hint at accelerated aging in smokers. Other mechanisms that may lead to enhancement of functional networks in the brains of smokers may more resemble addictive mechanisms: Complex alterations have previously been found in executive networks^13^, comprising vlPFC and SFG—structures that exhibited higher smoking-associated RSFC also in the current study. These alterations have been suggested to reflect a shift from more endogenous to more exogenous processing of addiction-related cues in smokers^13^. Smoking-related alterations in functional connectivity, however, seem not to be limited to frontal brain networks: Consistent with the altered RSFC between PMC and motor networks in the current study, disruptions in motor networks have previously been observed in smokers^19^, together with decreased GM density^12^. Smoking thus seems to be accompanied by a complex pattern of increases and decreases in functional connectivity affecting frontal and motor networks. Importantly, most of the here reported studies investigated smoking in young to middle-aged adults. Within the aged population, increase and dedifferentiation in RSFC seems to be one hallmark^29^. Smoking-related alterations in RSFC may therefore rather shift to systematic increases in RSFC than to decreases. Interestingly, while decreased functional coupling within motor networks has been previously observed in smokers in comparison to non-smokers^19^, RSFC amongst smokers was positively correlated with smoking severity. We also investigated smoking in a dose-dependent manner (the more pack years, the more RSFC), which may explain why we only found RSFC increases related to smoking and no systematic decreases (see Supplementary Figs. 1 and 2). Further, we found a systematic relation between smoking and dorsal occipital cortex (dOC, Fig. 3a, c, d, h, i), which is involved, for example, in visuospatial attention^57^. Influences of smoking on visuospatial attention have been circumscribed in terms of smoking facilitating visual attention by enhancing the blood oxygen-level-dependent (BOLD) signal in dorsal visual stream^58^ and higher coupling between visual processing regions^19^. These alterations in visual processing during acute nicotine withdrawal may shift towards altered RSFC patterns when abstaining from smoking. The increases were observed in networks related to sensory awareness and attention (dPMC and dOC), which may reflect a more general shift of sensory and attention systems towards addiction-related processing. Importantly, smoking was not related to structural decline in the current study, but was the only variable showing systematic associations to increased RSFC of those regions that showed decreased brain structure.

We hypothesized that genetic susceptibility may modify the relationship between lifestyle and the aged brain. Adjustment for genetic risk revealed an extra effect in the precuneus, indicating enhanced sensitivity of these analyses, while the general result pattern remained unchanged. Thus, genetic susceptibility seems to be a non-negligible, but not a strong contributor when it comes to the relationship between lifestyle and brain aging. Our approach shows that accounting for different concurrent influences may help to identify small effects. In future studies, it would be desirable to also include potential genetic influences of the other two lifestyle variables (social integration and physical activity), for which genetic factors had not been consistently identified at the time of our analysis.

Some further limitations of the current study should be addressed. First, the questionnaire items that were used to measure lifestyle variables concerned different time windows (e.g., physical activity within the last 4 weeks, smoking as number of cigarettes smoked over the whole lifetime). Each lifestyle variable was operationalized to be as representative as possible for the long-term lifestyle behavior of each person^59^. Epidemiological research has shown that assessments specifically regarding defined short time frames (e.g., a month, a week) are more reliable indicators of long-term behavior than self-reports regarding longer time frames (e.g., a whole year^60^). Future studies are warranted to additionally study the influence of changing lifestyle habits.

Additionally, all lifestyle habits were assessed using self-report, which may be influenced by memory effects or social desirability bias. However, self-report measurements have been shown to have high validity and reliability^60^ and are thus suitable in such an epidemiological population-based cohort setting.

Our cross-sectional and correlational design makes it impossible to determine causal directionality of effects or to rule out cohort effects. It is, for example, impossible to disentangle whether enhanced social integration leads to stronger cortical folding, or whether higher initial neuronal reserve leads to higher social integration. Further, it might be interesting to also include non-linear or differentially weighted effects when analyzing lifestyle variables individually or in combination. However, based on the current state of research, assumptions regarding a specific weighting of different lifestyle variables would be speculative. Future studies could use simulations to evaluate the potentially non-equal contributions of different lifestyle variables by simulating different weightings and examine their association to brain structure and function. This needs to be evaluated in future studies.

Based on the literature, we assumed the lifestyle behaviors to affect the brain in a certain direction, for example, negative effects of alcohol consumption on the brain. Effects in the opposite direction, depending on the brain region examined, may still occur. Importantly, the post-hoc multiple regressions using extracted cortical folding values confirmed our assumption of effect direction for the examined regions (Supplementary Figs. 8–9). Additionally, although our concept of social integration is widely used in epidemiological studies^22^, it covers mostly quantitative, summed-up measures of social network (visits of friends, relatives, or children). According to socio-emotional selectivity theory^61^, however, older adults select their companions based on relationship quality, leading to a small quantity of close friends rather than a large number of superficial acquaintances without feeling less emotionally supported. Future research could explore how the quality of social contacts contributes to brain aging.

Besides the known beneficial effects of physical activity^2–5^, our results emphasize the significance of low alcohol consumption and high social integration as protective factors in aging. Whereas interventions to preserve brain health based on physical activity are quite common^3^, research lacks randomized intervention trials of social integration of older adults, although both might be easily combined^8^. Further, other lifestyle variables such as dietary or sleeping habits should be examined in future studies.

In summary, our results provide insights into the complexity between environmental and genetic factors, brain structure, and functional connectivity of the aged brain. Our newly developed combined approach enabled us to examine this complexity and revealed that older individuals carrying higher lifestyle risk load seem to be at higher risk to suffer from structural brain atrophy. While these variations in structural decline were mainly explained by levels of alcohol consumption, physical activity, or social integration, smoking was the main variable driving the associations between higher lifestyle risk and increased RSFC. The increases in RSFC, though, may further reflect reduced cognitive reserve and accelerated aging related to smoking, as well as addiction-related functional adaptations of the brain. In consequence, a more protective lifestyle may contribute to brain reserve, that is, the preservation of brain structure, and to cognitive reserve, that is, the more efficient use of functional brain networks. Our study therefore shows that integrative concepts of lifestyle may be a strong instrument for advancing our understanding of risk and protective influences on aging in the general population and in patients suffering from neurodegenerative diseases, as well as for low-cost interventions preserving healthy aging.

## Methods

### Sample characteristics

Seven hundred and fifteen older adults from the population-based cohort of the 1000BRAINS study^21^, recruited from the Heinz Nixdorf Recall study^60^, were available for the current study. Two participants had to be excluded from analyses due to incidental findings. Seventy MR data sets could not be used for analyses of cortical characteristics due to poor quality of WM/GM segmentation (see MRI processing), surface reconstruction, or registration. Due to missing values in behavioral data 63 participants had to be excluded. After calculating the combined lifestyle risk scores (see Construction of lifestyle-related risk scores), we excluded another 31 participants as outliers (±3 SD from the mean) to ensure that these extreme values would not bias the overall outcome. Finally, 549 older adults (248 female) were included in the analyses. All participants gave written informed consent in agreement with the Declaration of Helsinki. The study protocol was approved by the Ethics Committee of the University of Essen, Germany. An overview of demographic data is given in Table 1.

### Materials

Lifestyle measures: Lifestyle data were retrieved from the database of the Heinz Nixdorf Recall study^59^.

Single lifestyle variables: Alcohol drinking behavior was assessed via a self-report questionnaire asking about average consumption of different beverages (beer, red wine, white wine, spirits, and cocktails) within the last 4 weeks. The proportion of pure alcohol per beverage was then multiplied with the frequency of drinking. Next, all beverages per person were summed up, resulting in the amount of total consumption of pure alcohol in grams per month (g/month).

The degree of lifetime exposure to tobacco smoking was assessed in pack years, calculated by multiplying the years of smoking with the self-reported number of smoked cigarettes per day (CPD).

Social integration was assessed using an adapted version of the social integration index developed by Berkman^22^. The present social integration index comprised three domains: The first domain represented “marital status.” Married or cohabitating participants were scored a 2; single, never married, widowed, or divorced participants were scored a 0. The second domain was “close ties” and represented a sum score of the number of children, close relatives, and friends reported by the participants. The third domain was “membership in organizations”. This domain represented a sum score of the number of organizations participants were members in and participated in at least once a month. Organizations included were: sport clubs, regional clubs, hunting clubs, choirs, theater clubs, music clubs, occupational or labor unions, political clubs or parties, congregations, and self-help groups. The scores of all three domains were summed up into the social integration score.

Physical effort was measured using the metabolic equivalent of task (MET^21^), a measurement for the energy expenditure of a given activity compared to rest. The compendium of physical activities^21^ provides a mean energy expenditure value per hour of each activity. It is based on several studies measuring energy expenditure of heterogeneous activities and lists activities, which include willful physical exercise, but also several physically stressful activities not intending exercise, like cleaning or home carpentry. Participants were asked to report up to four different sportive (e.g., running) and up to four different physical activities (e.g., gardening), carried out within the last month. Based on the MET values assigned to the activities listed in the compendium, MET values were assigned to each of the activities reported by the participants and multiplied with the duration in hours (per month). Finally, a sum score of all activities was built.

Construction of the combined lifestyle risk score: Based on the literature, we classified cigarette smoking^11,13,19^ and alcohol consumption^15–18^ as risk variables for brain atrophy. Social integration^1,6,7,50^ and physical activity^2–5^ in contrast were classified as protective variables. Raw data on each single lifestyle variable were transformed into *z*-scores to obtain uniform measure units. The first aim was to obtain a risk score that indicated higher risk in higher values. Therefore, we reversed signs of the protective variables (social integration and physical activity), such that negative values reflected higher protection. The second aim was to obtain a risk score where a value of zero would indicate a mathematical balance of negative and protective behavior. Hence, we applied an additional linear transformation on the *z*-transformed lifestyle variables before summing them up: The protective variables were linearly transformed into negative values (by subtracting the maximum value from each value). Analogously, the risk variables were linearly transformed into positive values (by adding the minimum value to each value). Hence, all values for risk variables were positive. Finally, the linearly transformed values of all lifestyle variables were summed up into one combined lifestyle risk score, which indicated protection as negative values and risk as positive values.

Stepwise exclusion of single lifestyle variables: To examine the contributions of each lifestyle variable to the combined lifestyle risk score, we used a stepwise approach to exclude single lifestyle variables from the risk score. This resulted in four risk score models integrating three single lifestyle variables, and another four risk score models including only two single variables (Supplementary Table 1). The first possible exclusions are exemplarily described here: For example, we first excluded social integration from the combined lifestyle risk score, resulting in a model including physical activity, alcohol consumption, and smoking (Fig. 2b). In the next step, we additionally excluded smoking from the risk score, which now included only physical activity and alcohol intake (Fig. 2f).

Genetic data: Lymphocyte DNA from participants was isolated from ethylenediaminetetraacetic acid-coagulated venous blood by a Chemagic Magnetic Separation Module I (Chemagen, Baesweiler, Germany). DNA samples were genome-wide genotyped using Infinium assays (Illumina, San Diego, CA, USA) for BeadChips HumanOmniExpress, HumanOmni1-Quad, or HumanCoreExome. Quality control of raw genotype data comprised an exclusion of single-nucleotide polymorphisms (SNPs) (deviation from Hardy–Weinberg equilibrium (HWE): *p* ≤ 1 × 10^–4^; genotyping call rate: ≤95%; minor allele frequency: MAF ≤ 3%) and participants (SNP-based principal component analysis: >8 SD from the mean in one of the first ten principal components; mismatch between self-reported and X-chromosomal-derived gender). To increase the number of available SNPs and decrease the number of missing genotype calls, dosage data were generated for all participants using IMPUTE (version 2.3.1) as tool and phased haplotypes from The 1000Genomes Project (ALL macGT1 reference panel, phase 1, release 3, March 2012) as reference.

The next step was the identification of phenotypically relevant SNPs that would be included into the PRS. To further test whether genetic factors modify the association between lifestyle risk and variations in cortical folding as well as functional connectivity of older adults, we reviewed recent literature on common genetic factors for lifestyle risk in European ancestry populations. Study selection was based on the NHGRI-EBI Catalog of published GWAS as of 23th May 2018, provided by the National Human Genome Research Institute (NHGRI) of the United Kingdom and the European Bioinformatics Institute (EMBL-EBI) available at www.ebi.ac.uk/gwas.

First, we identified studies that examined quantitative traits matching those we investigated in the current study, namely smoking and alcohol consumption as risk variables, and physical activity and social integration as protective variables. Concerning physical activity and social integration, there were no GWAS examining a matching phenotype in European ancestry populations. Further, there were no studies available investigating the genetic basis of lifetime smoking operationalized as pack years, but as in CPD, which was the closely related phenotype found. Concerning the risk variables of alcohol consumption, due to the population-based nature of our study, we found studies investigating alcohol consumption in population-based cohorts and not in clinical cohorts. Secondly, from those studies investigating one of the two phenotypes, we chose only those that found significant association at genome-wide significance (*p* value <5 × 10^−8^). Additionally, we chose only those for which results had been replicated in a second sample of European ancestry. By doing so, we identified three major studies of interest for the phenotype “smoking” (in CPD)^31–33^, and one study for “alcohol consumption”^34^. Within the three publications relevant to smoking, the SNP rs1051730 was identified as the top SNP for smoking quantity in CPD; ten additional SNPs also showed significant association with CDP. For alcohol, the number of candidate SNPs was 14. From the overall set of 25 SNPs, we included those meeting the following conditions: Small difference of MAF (≤3%) between the reference population (1000Genomes CEU) and the current 1000BRAINS study, no strong linkage disequilibrium (<0.8) to other selected SNPs of this phenotype, and high imputation quality (median info score = 99.5%), as well as no deviation from HWE (*p* > 0.05) in the subsample of 1000BRAINS. Finally, this resulted in nine SNPs for the phenotype “smoking” and seven SNPs regarding the phenotype “alcohol consumption” (Table 3). In the next step, genetic information provided by the quality controlled 16 SNPs was transformed into a PRS.

PRS was calculated using the weighted allelic scoring routine by PLINK (v1.9). In particular, effect alleles and effect sizes (*R*^2^) were used as defined by the original studies (Table 3). The individual GRS value was then calculated as the mean of the summarized effects in an SNP set that is for the SNP set of alcohol consumption and for the SNP set of CPD. This resulted in individualized values of combined genetic risk for smoking and alcohol consumption for each participant of the current study.

Acquisition and processing of structural MR images: T1-weighted anatomical three-dimensional (3D) images were collected with a 3 T Tim-TRIO MR scanner (Siemens Medical System, Erlangen, Germany). The following scan parameters were used: repetition time = 2.25 s, echo time = 3.03 ms, inversion time = 900 ms, field of view = 256 × 256mm2, flip angle = 9°, voxel resolution = 1 × 1 × 1 mm^3^, 176 axial slices. A detailed description of the 1000BRAINS study protocol can be found in Caspers et al.^20^.

3D images were processed using the automated surface-based pipeline of the FreeSurfer Software package (version 5.3.0, Athinoula A. Martinos Center for Biomedical Imaging). A detailed description of all steps included in the streamline was provided by Dale et al.^27^ and Fischl et al.^62^ and in the FreeSurfer documentation at http://surfer.nmr.mgh.harvard.edu. Processing includes motion correction, intensity normalization, removing of extra-cerebral voxels (non-brain tissue) using SPM12 (The Wellcome Dept. of Imaging Neuroscience, London; www.fil.ion.ucl.ac.uk/spm), spatial normalization, volumetric segmentation^62^, and cortical surface reconstruction^27,63^. To reconstruct the cortical surface, first the so-called white surface is generated at the interface of WM and GM. Then, the pial surface is created at the interface between GM and the cerebrospinal fluid (CSF). The final mesh model of the pial surface is tessellated into triangles and consists of about 120,000 vertices per hemisphere with an average surface area of 0.5 mm^2^.

Vertex-wise LGI was calculated using the surface-based approach as implemented in FreeSurfer^26^, which is the 3D extension of the work by Zilles et al.^23^: First, an outer hull of the pial surface (outer smoothed surface) is created. This is obtained by a morphological closing operation and follows the exposed (visible) surface along the gyri but does not reach into the segments buried within the sulci. Then, for each 100th vertex on the outer surface the ratio between the area on the pial surface and the corresponding area on the outer smoothed surface (both defined as a circular region with 25 mm radius around the vertex^26^) was calculated. Using a weighted average, finally local gyrification indices were calculated at each vertex.

CT was also extracted using FreeSurfer^28^. First, the boundary between GM and WM was identified. CT was then measured by finding the shortest distance between a given point on the reconstructed pial surface and the GM/WM boundary surface and vice versa^28^. Finally, averaging both values resulted in about 150,000 CT values per hemisphere.

Subcortical structures were segmented using the automatic segmentation provided by FreeSurfer^63^. Here, subcortical GM is automatically segmented into different volumes. Then, a neuroanatomical label is assigned to each volume based on probabilistic information estimated from a manually labeled data set.

Acquisition and processing of functional MR images: We investigated functional connectivity as measured by resting-state functional MRI. BOLD signal time series were acquired using gradient-echo echo planar imaging (EPI) pulse sequences (300 images, TR 2.2 s, 36 axial slices^20^). The first four images were discarded and the remaining images were processed using SPM12. Head motion correction was done by affine registration using a two-pass procedure registering all images to the individual mean of the respective participant. This mean image was spatially normalized to MNI152 using the unified segmentation procedure^64^. We then applied the resulting deformation to the individual EPI volumes. Residual anatomical variations were compensated for by smoothing with a Gaussian kernel of 5 mm full width at half maximum, which additionally approximates requirements of normal distribution of the residuals for Gaussian random field inference to correct for multiple comparisons^65^. Variance that could be explained by first- or second-order effects of the following covariates was removed for each voxel’s time series: (i) the six motion parameters derived from the image realignment; (ii) their first derivative; (iii) mean GM and WM as well as CSF signal intensity. The first three covariates (i–iii) entered the model as first- and second-order terms, which was shown to increase specificity and sensitivity of the FC analyses^65^. Finally, the data were band pass filtered between 0.01 and 0.08 Hz to keep only those frequencies most relevant for studying neural signal fluctuations in the brain^66^.

For analyses of differences in RSFC, the maximum vertices of the structurally localized regions in dPMC and vlPFC were determined and transformed into MNI space. A sphere of 5 mm was then drawn around the coordinates and used as seed volumes of interest (VOIs). The time courses of these VOIs were extracted for each participant as the first eigenvariate of all GM voxels according to segmentation within the respective VOI, mainly since this eigenvariate is robust against inter-individual variance in anatomical localization and distribution of voxels mainly driving the functionally relevant and representative VOI time series used for FC analysis to a certain extent^65^.

Statistical analyses of surface-based measures: All statistical analyses regarding the association between combined lifestyle risk and local gyrification, as well as CT were carried out using Qdec, a graphical user interface implemented in the FreeSurfer software package (http://surfer.nmr.mgh.harvard.edu) and IBM SPSS Statistics 20.0. General linear models (GLMs), as implemented in Qdec, were used to evaluate the association between lifestyle risk and vertex-wise LGI, as well as CT, respectively. Qdec allows whole-brain analyses of surface morphology, thus no specific brain region needs to be defined a priori to test for an effect of behavioral data. Instead, each vertex on the cortical surface is tested for an association between the parameter of interest (cortical folding, CT) and the behavior of interest (lifestyle). Here, we calculated a linear regression using the risk score as explanatory variable and LGI and CT as dependent variables. Gender and age were used as covariates that were statistically controlled for. Two-tailed *F* tests were used to test whether lifestyle risk would be associated with higher or lower LGI and CT. Corrections for multiple comparisons were performed by testing results against a simulated null distribution of maximum cluster size across 10,000 iterations using Monte Carlo Z simulation as implemented in Qdec^67^ using a cluster-forming threshold of *α* = 0.05. This analysis was then recalculated with the inclusion of the PRS as a third covariate, as well as with depressive symptomatology, as measured with the BDI-II^36^, ISCED^37^.

To specifically describe the association between combined lifestyle risk and variations in local cortical folding, we extracted LGI values post hoc at the cluster’s maximum vertex of those regions that showed variations in cortical folding associated with combined lifestyle risk in the main analyses. To estimate the contributions of the single lifestyle variables to cortical folding of these regions with a different technique than the stepwise exclusion procedure, these LGI values were imported into IBM SPSS Statistics 20.0 and submitted as dependent variable into a multiple linear regression using the remove method. All four single lifestyle variables, age, and gender were submitted as explanatory variables to the first model. Then, single lifestyle variables were removed step by step from this model, while changes in *F* and *R*^2^ were measured for each step. When submitting the extracted cortical folding values of the dPMC as dependent variable to the first model, we excluded physical activity in the first step and alcohol consumption in the second. This was done because the stepwise exclusion procedure in our main analysis hinted at these two variables as the main contributors to cortical folding in this region. Third, social integration and fourth, pack years were excluded. Regarding the vlPFC, the stepwise exclusion procedure hinted at social integration as the main contributor to differences in cortical folding. Therefore, we excluded social integration in the first step when using the extracted cortical folding values of the vlPFC as dependent variable. Second physical activity, third alcohol consumption, and fourth pack years were excluded.

We then applied our stepwise exclusion procedure. All models derived from the stepwise exclusion procedure were tested in the same manner as the combined lifestyle risk score using linear regression models with gender and age as covariates that were statistically controlled for. Again, all results were corrected for multiple comparisons using Monte Carlo Z simulation with *α* = 0.05 and a cluster-wise *p*-value <0.01.

We examined the effects of single lifestyle variables on LGI and CT, using a series of GLMs: For each linear model, *z*-transformed data of one lifestyle variable (alcohol consumption, smoking, social integration, physical activity) was taken as the predictor. Comparable to the overall lifestyle risk score analysis, we included age and gender as covariates that were statistically controlled for and tested two-sided *F* tests. These GLMs were corrected for multiple comparisons by testing results against permuted data in 10,000 iterations at *α* < 0.05 and a cluster-wise *p*-value <0.01, as implemented in FreeSurfer. We chose to use permutation testing, because data of single lifestyle variables showed a highly skewed distribution and permutation-based inference as an exact non-parametric statistical test is applicable to skewed data^68^.

Statistical analyses of subcortical structures: To examine the association between GM volume of subcortical structures and lifestyle, we imported individual volumes of 20 subcortical structures into SPSS and calculated partial Spearman's correlations between the combined lifestyle risk score, the different risk score models and single lifestyle variables, controlling for age, gender, and total intracranial as covariates of non-interest. We additionally applied a post-hoc Bonferroni correction using a threshold of *α*_corr_ = 0.05/20 = 0.0025 with 20 subcortical structures (Table 2) tested.

Statistical analyses of RSFC: To assess the association between combined lifestyle risk and RSFC we computed linear (Pearson's) correlation coefficients between the extracted time courses of the seed regions derived from the analysis of differences in local brain structure, namely the dPMC and the vlPFC, and the time series of all other GM voxels in the brain. The voxel-wise correlation coefficients were then transformed into Fisher’s *Z*-scores, and tested for consistency across subjects by a second-level multivariate analysis of variance (including appropriate non-sphericity correction) with the combined lifestyle risk score as explanatory variable (linear regression model). Results are reported at *α* = 0.05 and tested two-sided for increases, as well as decreases in RSFC (cluster level corrected, cluster-forming threshold *α* = 0.001).

Comparable to the surface-based analyses all models derived from the stepwise exclusion procedure were tested for associations between the two seeds and RSFC of all GM voxels in the same manner as the combined lifestyle risk score using linear regression models while statistically controlling for gender and age as covariates. Again, all results were thresholded at an uncorrected *α* value of *α* = 0.001 (cluster-forming threshold) and corrected at the cluster level at *α* = 0.05.

The same analyses conducted for the combined lifestyle risk score, as well as the different risk score models derived from the stepwise exclusion procedure, were also applied to investigate the association between single lifestyle variables and RSFC of the two seeds. Here, always one single lifestyle variables was investigated at one time.

Additional adjustment for polygenic risk: To see whether genetic influences would modulate the relation between combined lifestyle risk scores and the aging brain, we calculated the analyses of gyrification, CT and the analyses of RSFC twice: (i) Without further adjustment for GRS and (ii) with additional adjustment for GRS. Please note that all analyses were corrected for age and gender.

Sensitivity analyses: Subsequent to the main analysis, we conducted additional sensitivity analyses as often used in epidemiological research to confirm our results. From each single lifestyle variable we calculated residuals, corrected for the three other single lifestyle variables: for example, we calculated residuals for social integration by correcting for physical activity, alcohol consumption, and smoking using partial correlations. The purpose was to clean each lifestyle variable from any variance introduced by the other three lifestyle variables and to test whether the results of the main analyses could be replicated. We then used these residuals to again calculate the combined lifestyle risk score. Further, we repeated the stepwise exclusion in the same manner as in the main analyses.

Anatomical allocation of significant findings: Significant clusters resulting from the surface-based as well as the RSFC analysis were anatomically interpreted using the JuBrain Cytoarchitectonic Atlas^69^. Concerning the surface-based analysis, we converted coordinates of significant surface-based clusters from Talairach to MNI space using the transformation tool “mri_surf2vol” as provided by FreeSurfer. Concerning the RSFC analysis, the thresholded statistical parametric maps resulting from the analyses on the different risk scores were used. Overlap between significant clusters and cytoarchitectonically defined areas was determined using the SPM Anatomy toolbox 2.2c^70^ available at http://www.fz-juelich.de/inm/inm1/DE/Forschung/_docs/SPMAnatomyToolbox/SPMAnatomyToolbox_node.html. This was done using SPM12 (The Wellcome Dept. of Imaging Neuroscience, London; www.fil.ion.ucl.ac.uk/spm) within the environment of Matlab (The MathWorks Inc., Natick, MA, USA). Localization of significant clusters is therefore given on a macroanatomical and a cytoarchitectonic level where available (see Supplementary Tables 2–12).

## Supplementary Information

Suppmentary Information

Supplementary Data 1

Description of Additional Supplementary Files

## Data Availability

The data sets generated and/or analyzed during the current study will be made available from the corresponding author to other scientists on request in anonymized format and according to data protection policy in the ethics agreement.
