# Supplementary material for: Combining lifestyle risks to disentangle brain structure and functional connectivity differences in older adults
Source: Nat Commun. 2019 Feb 6;10:621. doi: 10.1038/s41467-019-08500-x (PMC6365564; doi:10.1038/s41467-019-08500-x)
Supplement: Supplementary file 1 — Suppmentary Information [file 41467_2019_8500_MOESM1_ESM.docx]

**Supplementary Information**

Bittner et al. “Combining lifestyle risks to disentangle brain structure and functional connectivity differences in older adults”

**Supplementary Methods**

**Associations between lifestyle risk and cortical folding: Post-hoc multiple linear regression within IBM SPSS Statistics 20.0**.

***Associations between lifestyle risk and cortical folding.*** Higher combined lifestyle risk was associated with lower cortical folding in two distinct cortical areas (Figure 2a): First, left dorsal premotor cortex (dPMC, *p* = 0.0001) and second, ventro-lateral prefrontal cortex (vlPFC, *p* = 0.0001) extending from the frontal pole to the middle frontal sulcus and to the posterior portions of the inferior frontal gyrus and sulcus. The stepwise exclusion approach hinted at alcohol consumption and physical activity being the driving variables behind this association as it disappeared when excluding both, alcohol consumption and physical activity (Figure 2i) from the risk score models.

To confirm this, we extracted cortical folding values within the dPMC and imported them to IBM SPSS Statistics 20.0 and submitted them as dependent variable in a multiple linear regression using the “remove” method. All four single lifestyle variables, age, and gender were submitted as explanatory variables to the first model. We used the remove method such that removing specific variables would reveal the individual contribution to the specific pattern, while change in F and r^2^ for each step was measured.

Regarding the extracted cortical folding of the dPMC, the first model explained 2.9% of the variance in cortical folding of the dPMC, *F* (1,542) = 3.69, R^2^ = 0.029, *p* = 0.0013. Excluding physical activity reduced the explained variance to 2% and resulted in a significantly lower model fit, *F*(1,542) = 3.37, R2 = 0.02, *F*-change = 5.64, *p* = 0.018. Excluding alcohol consumption in the second step did not result in a significantly different model fit (R^2^ = 0.017, *F*-change = 2.63, *p* = 0.106), while the overall model was still significant (*F*(1,543) = 3.42, *p* = 0.009). In the next step social integration was removed, which didn´t lead to a significantly different model fit (R^2^ = 0.015, *p* = 0.133, *F*-change = 2.27), while the model was not significant anymore (*F*(1,544) = 3.79, *p* = 0.103). When last excluding pack years, the model explained significantly less variance (R^2^ = 0.008, *p* = 0.027, *F*-change = 4.95), but was significant (*F*(1,545) = 3.19, *p* = 0.042). Hence, physical activity indeed explained the greatest amount of variance amongst the lifestyle variables as suggested by the stepwise exclusion procedure in the main analysis. Here, exclusion of alcohol consumption did not change the amount of explained variance significantly in contrast to the stepwise exclusion procedure.

Submitting the extracted cortical folding values of the vlPFC as dependent variable, the first model explained 3.1%, *F*(1,542) = 3.95 (R^2^ = 0.031, *p* = 0.0007). Excluding social integration reduced the explained variance to 2% and resulted in a significantly lower model fit (*F*(1,542) = 3.25 (R^2^ = 0.02, *F*-change = 7.26, *p* = 0.007). Excluding physical activity in the second step did not result in a significantly lower model fit (R^2^ = 0.018, *F*-change = 1.996, *p* = 0.158,), while the overall model was still significant (*F*(1,543) = 3.555, *p* = 0.007). In the next step, alcohol consumption was removed, which didn´t lead to a significantly different model fit fit (R^2^ = 0.02, *p* = 0.835, *F*-change = 0.044), while the model was still significant, *F*(1,544) = 4.733, p = 0.003). When last excluding pack years, the model explained significantly less variance (R^2^ = 0.013, *p* = 0.024, *F*-change = 5.16). In summary, this multiple linear regression revealed social integration as the most contributing variable to the differences in cortical folding in vlPFC. Beta weights for each single lifestyle variable as estimated for the first model including sex, age, and all four single lifestyle variables are shown in Supplementary Figure 9.

**Associations between lifestyle risk and cortical folding: Additional systematic reductions in cortical folding observed in the stepwise exclusion procedure.** The stepwise exclusion models yielded additional systematic reductions in cortical folding (Fig. 2, Suppl. Table 2): Decreased cortical folding in right dPMC, expanding to primary motor cortex, was associated with higher lifestyle risk in the models that included a combination from physical activity, alcohol consumption, or social integration (Fig. 2c, 2f, 2g). Decreased cortical folding in left frontal pole was found for the risk score models that included different combinations of social integration (Fig. 2d, 2g, 2i).

**Sensitivity analyses**

As sensitivity analysis, we calculated residuals from each single lifestyle variable, corrected for the three other single lifestyle variables (see methods) to clean each lifestyle variable from any variance influenced by the other three lifestyle variables. We calculated the combined lifestyle risk score from these residuals again and repeated the stepwise exclusion similar to the main analyses for replication purposes. Results of the sensitivity analyses reproduced almost entirely the result pattern of the main analyses of the association combined lifestyle risk, cortical folding and RSFC. An additional association was only found between the combined lifestyle risk score and cortical folding in the right dorsal premotor cortex (dPMC, Suppl. Fig. 6a). Compared to the main analyses, the clusters found in the right homologue of the dPMC were larger and expanding more into posterior parts of the precentral gyrus (Suppl. Fig. 6c, f, g). Exact anatomical localization is given in suppl. Table 10. Regarding RSFC, we still found the same pattern in the results: Risk score models including smoking showed higher RSFC between the dPMC and the sensorimotor cortex and between the vlPFC and the superior frontal gyrus, respectively. The sensitivity analyses, though, revealed additional significant associations: The risk score model composed of physical activity, alcohol consumption and social integration, as well as the risk score model including alcohol consumption and social integration, showed higher RSFC between the dPMC and the left hippocampus (Suppl. Fig. 7c & g). Further, alcohol consumption as a single variable showed additional associations between the dPMC and large clusters in the inferior temporal lobe and several subcortical nuclei (Figure 7k). Compared to the main analysis, the combined lifestyle risk score showed no significant association to increases in RSFC of the vlPFC. Still, the main pattern of risk score models including smoking being significantly associated to increased RSFC of the vlPFC was found (Suppl. Fig. 8).

Exact anatomical localization is given in suppl. Table 11 and Table 12.

**Associations between lifestyle risk and cortical folding: Additional adjustment for non-lifestyle variables.** Additionally, adding BDI-II^1^, ISCED^2^, or both, respectively, as covariates did not change the general association between combined lifestyle risk and cortical folding (Figure 6). However, in the stepwise exclusion approach some of the risk score models did not reach significance - particularly those risk score models, which were associated with left dPMC, while those associated with right vlPFC remained largely unaltered when applying different sets of covariates (Suppl. Fig. 11 – 13). It is particularly interesting that this association with social integration and right VLPFC survived even if depressive symptomatology is now regressed out. This further supports the strong association which was already consistently found in our original analysis. Contrarily, the association to left dPMC was not that stable, as already evident from our original analysis. Since it was only found in risk score models including both alcohol consumption and physical activity, depressive symptomatology seemed to additionally contribute to this more complex association.

As there were only changes within the extend, but not the location of the brain regions associated to lifestyle risk when additionally correcting for depressive symptomatology and / or depressive symptomatology, respectively, we did not provide additional Tables presenting the associated cytoarchitectonically defined areas.

**Supplementary analysis of associations between lifestyle risk and cortical thickness:** To complement our analysis of lifestyle risk and cortical folding, we performed supplementary analyses of an association between combined lifestyle risk and vertex-wise cortical thickness as an additional dimension in surface-based analyses^3^.

Within the analysis of cortical thickness, we again found a reoccurring association between those risk score models integrating alcohol consumption and physical activity and the left dPMC (Suppl. Fig. 14a, b, c, e & f). Further, risk score models including alcohol consumption, and / or smoking were associated with decreased CT in left inferior and superior parietal lobule. Uncorrected results can be found in Suppl. Fig. 15 & 16. No association between lifestyle risk and CT of right vlPFC could be found. It might thus be assumed that the so-far strong association between lifestyle risk and brain structure and right vlPFC, which is also highly robust against confounders, might be attributable to other mechanisms involving curvature and cortical folding as compared to the effects on CT.

**Supplementary Figures**


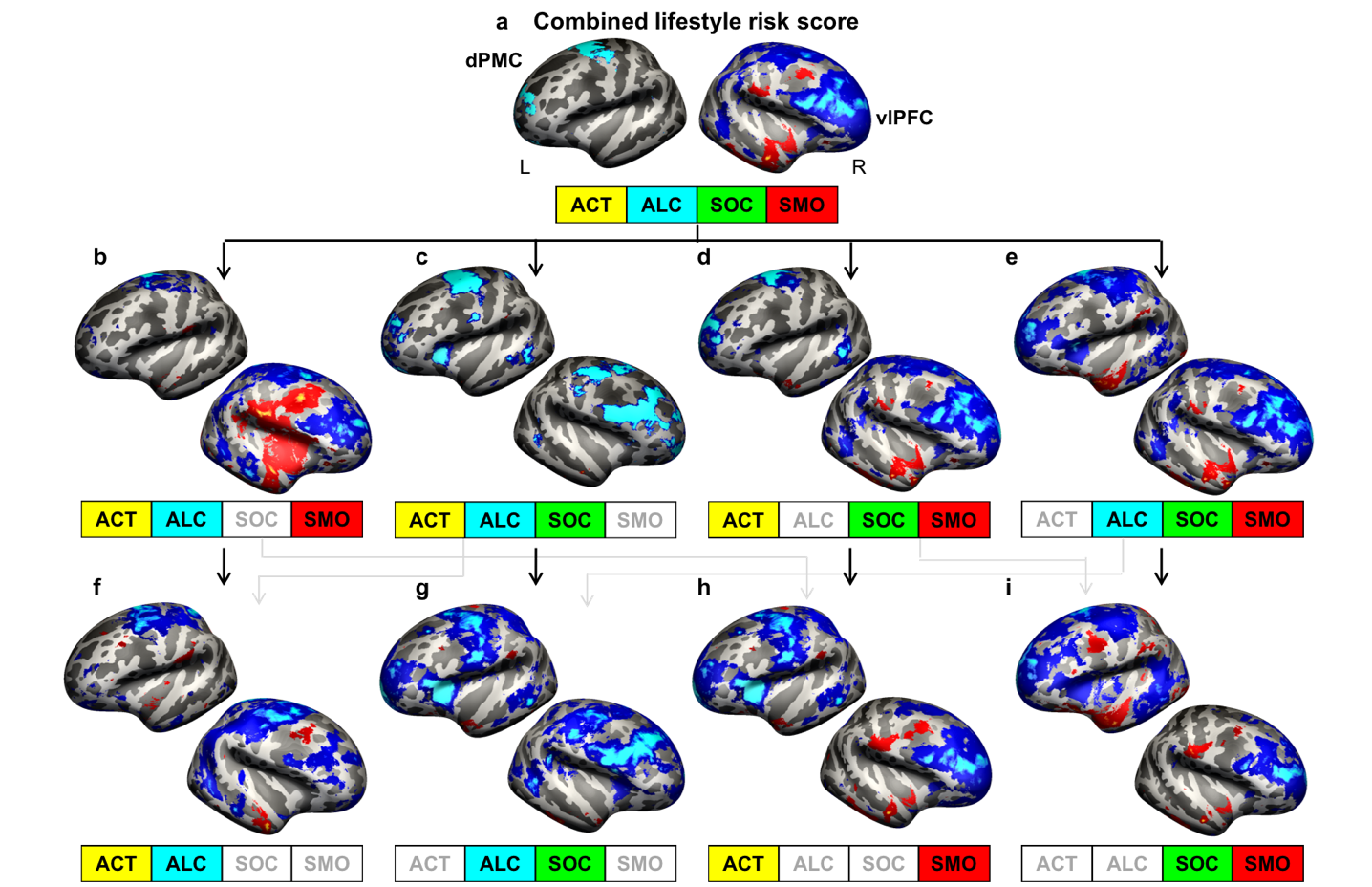


**Supplementary Figure 1: Brain regions showing alterations in cortical folding associated with lifestyle risk without correction for multiple comparisons.**

Associations between different risk score models and cortical folding without any correction for multiple comparisons depicted on the inflated surfaces of the fsaverage brain. Red colours indicate a positive association, while blue colours indicate a negative association between lifestyle risk and cortical folding.

Abbreviations: ACT = physical activity, ALC = alcohol consumption, SOC = social integration, SMO = pack years of smoking, L = left hemisphere, R = right hemisphere.

**
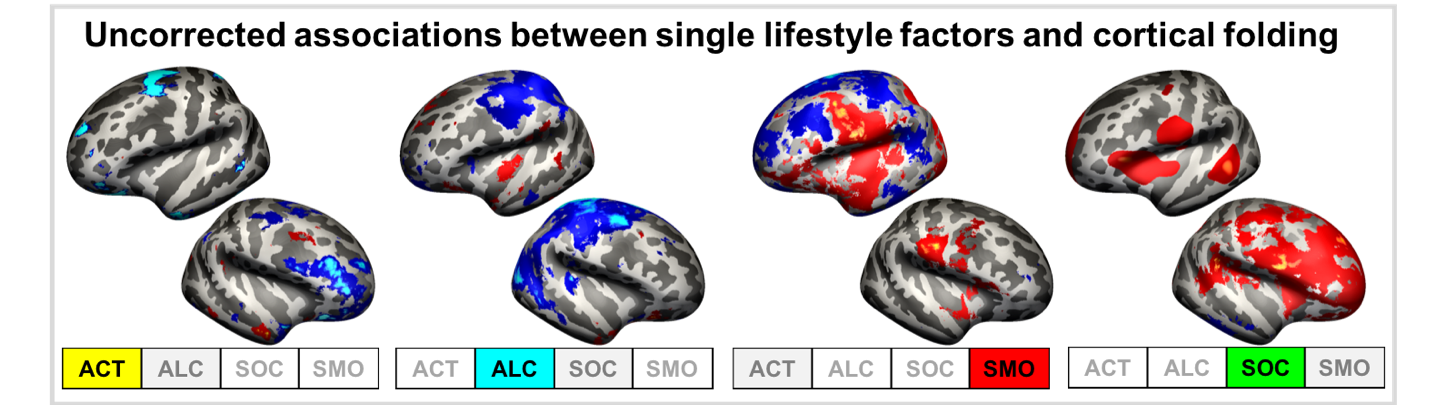
**

**Supplementary Figure 2: Brain regions showing alterations in cortical folding associated with all four single lifestyle variables without correction for multiple comparisons.**

Associations between single lifestyle variables and cortical folding without any correction for multiple comparisons depicted on the inflated surfaces of the fsaverage brain. For further conventions and abbreviations please see Suppl. Fig. 1.

**
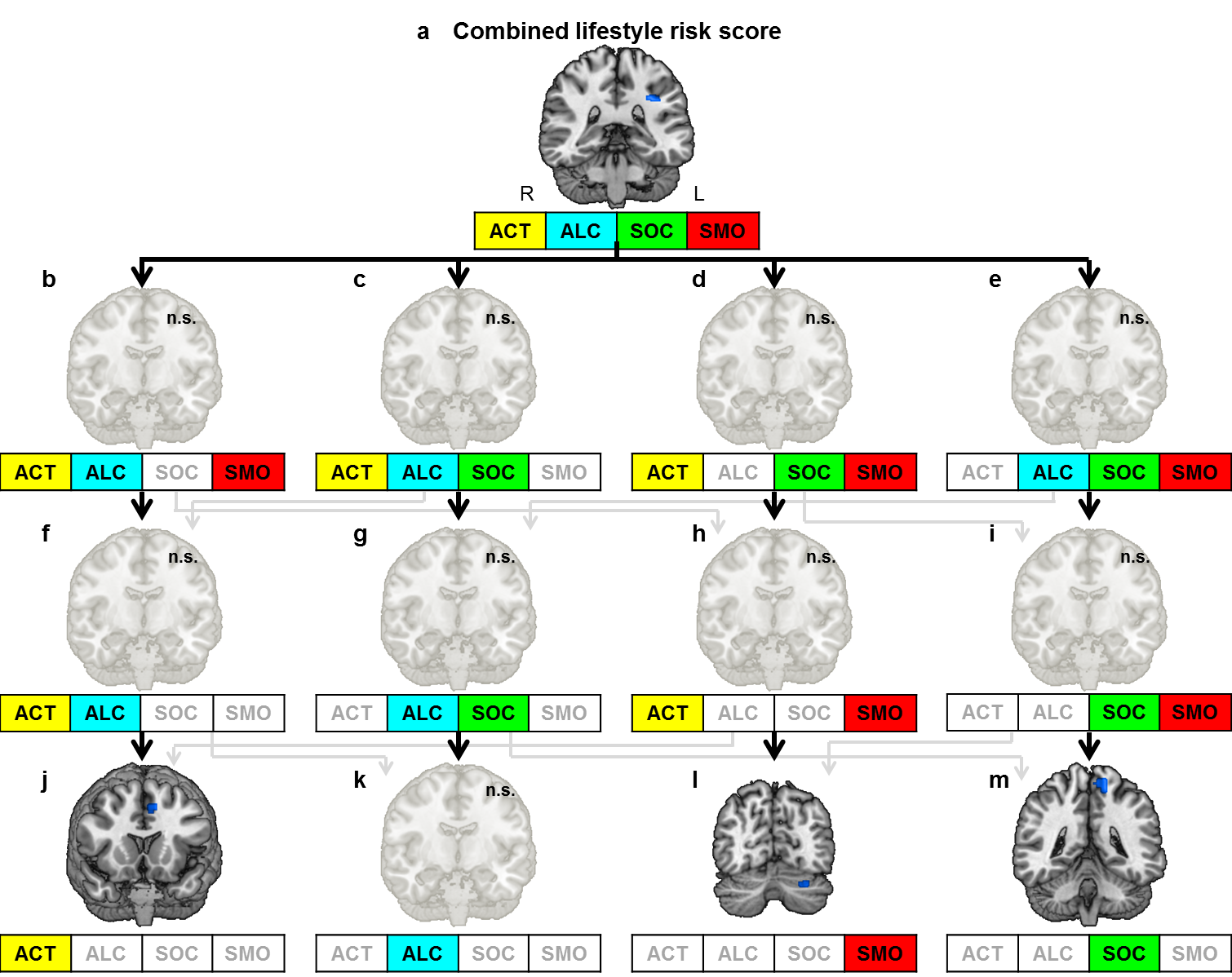
**

**Supplementary Figure 3: Brain regions showing a lifestyle risk associated decrease in RSFC to the seed in the left dPMC.**

Abbreviations in the small boxes refer to the same meaning as in Figure 2. Coronar sections show decreases in RSFC depicted in blue. n.s. = not significant, dPMC = dorsal PMC, vlPFC = ventro-lateral prefrontal cortex.

**
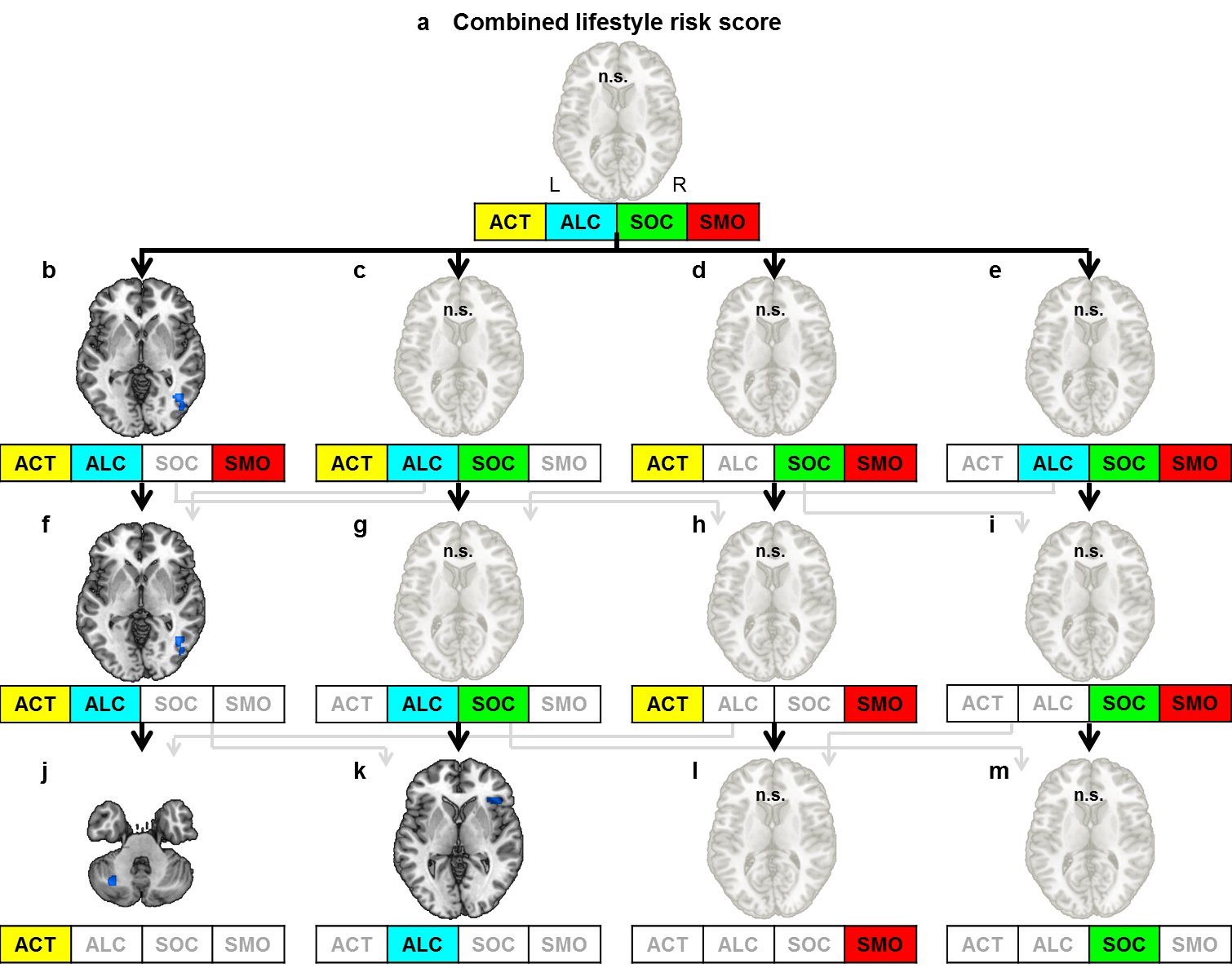
**

**Supplementary Figure 4: Brain regions showing a lifestyle risk associated decrease in RSFC to the seed in the right vlPFC.** Abbreviations in the small boxes refer to the same meaning as in Figure 2. Transversal sections show decreases in RSFC depicted in blue. n.s. = not significant, dPMC = dorsal PMC, vlPFC = ventro-lateral prefrontal cortex.

**
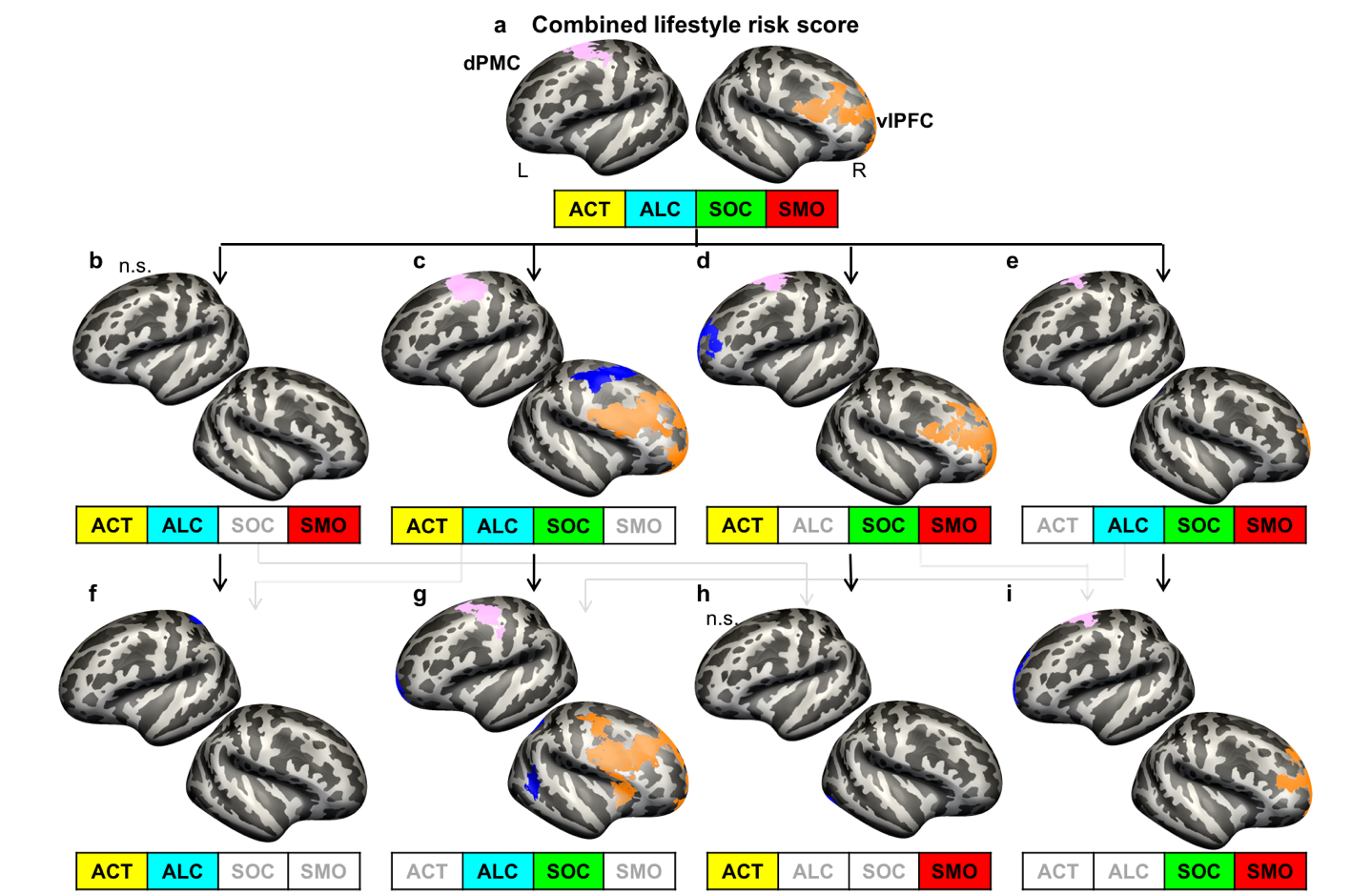
**

**Supplementary Figure 5: Brain regions showing alterations in cortical folding associated with lifestyle risk when additionally adjusting for polygenic risk.**

Associations between different risk score models and cortical folding when additionally adjusting for genetic risk as measured with the polygenic risk score (PRS). All results are depicted on the inflated surfaces of fsaverage. The recurrent associations between higher lifestyle risk and reduced cortical folding in the left dorsal premotor Cortex (PMC) and the right ventro-lateral prefrontal Cortex (PFC) are highlighted in rose and orange, respectively. n.s. = not significant, dPMC = dorsal PMC, vlPFC = ventro-lateral prefrontal cortex.

**
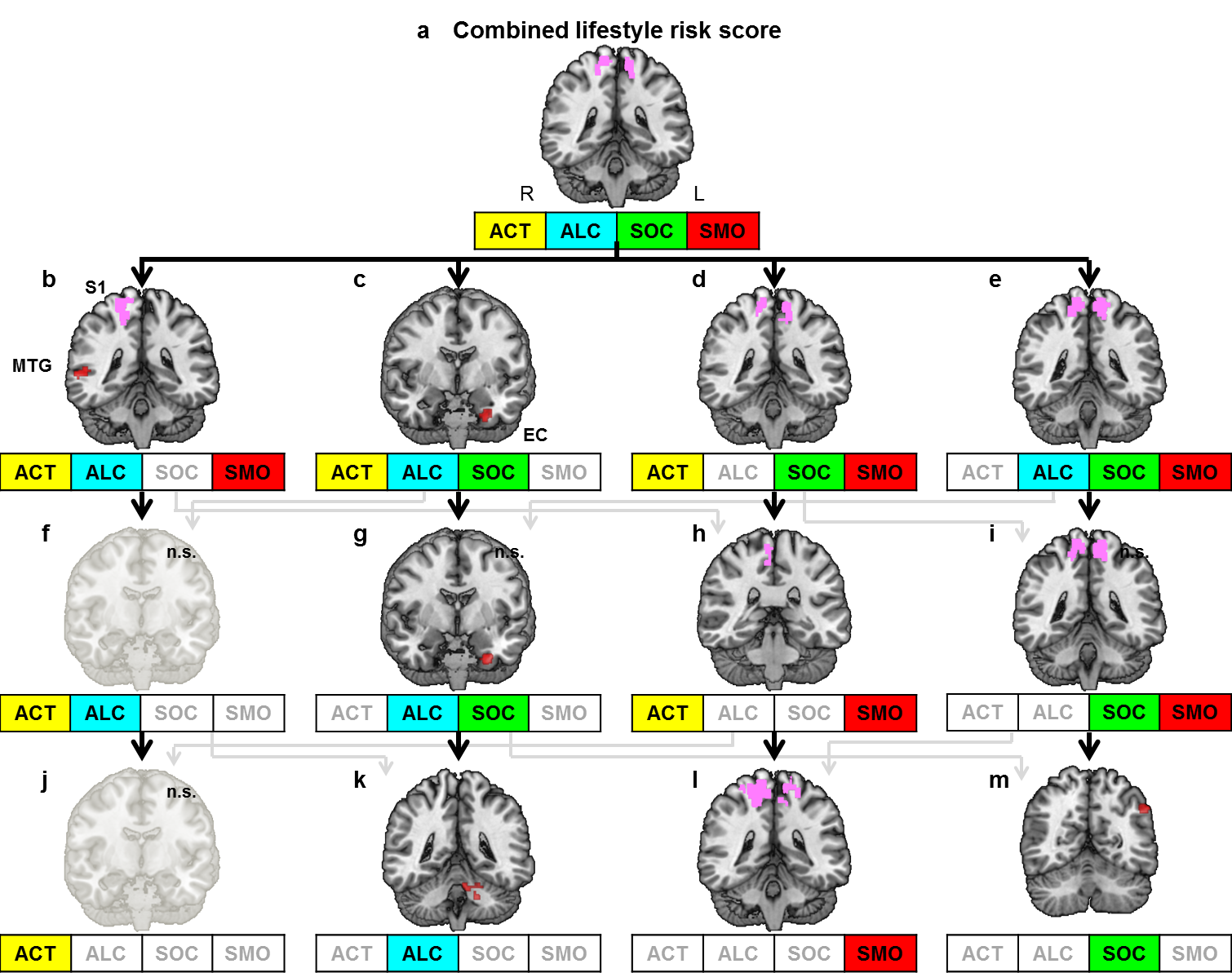
**

**Supplementary Figure 6:** **Brain regions showing a lifestyle risk associated increase in RSFC to the seed in the right dPMC when additional adjusting for polygenic risk.**

Figure depicts the associations between different risk score models and brain regions showing increased RSFC to the seed in dPMC in relation to higher lifestyle risk when additionally adjusting for genetic risk as measured with the combined genetic risk score (GRS). Abbreviations are explained in Supplementary Figure 1.

**
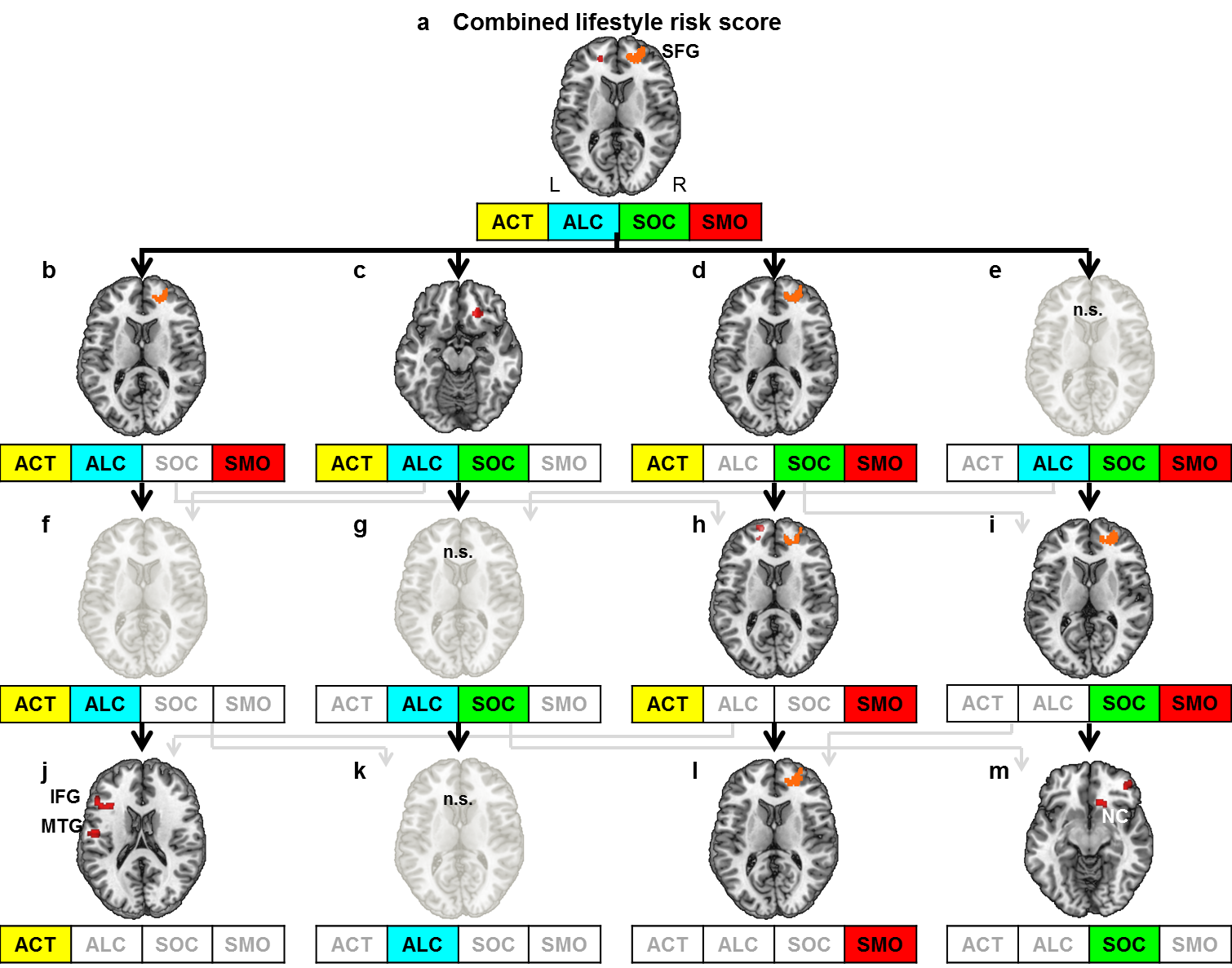
**

**Supplementary Figure 7:** **Brain regions showing a lifestyle risk associated increase in RSFC to the seed in the right vlPFC when additional adjusting for polygenic risk.**

Associations between different risk score models and regions showing increased RSFC to the seed in vlPFC in relation to higher lifestyle risk when additionally adjusting for genetic risk as measured with the combined genetic risk score (PRS) are shown. Explanations of abbreviations can be found in Supplementary Figure 1.

**
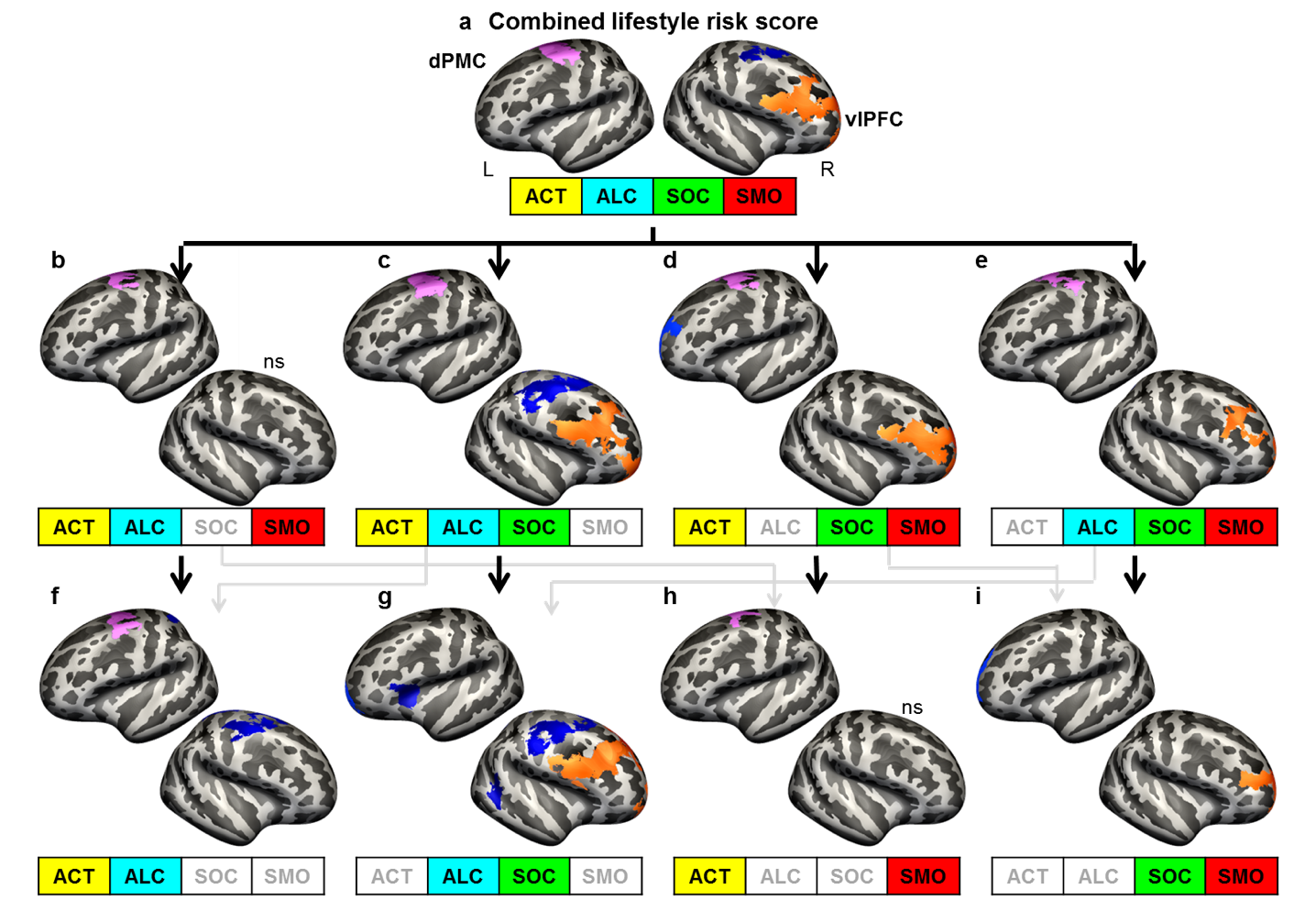
**

**Supplementary Figure 8: Sensitivity analysis for the association between lifestyle risk and cortical folding.**

The figure depicts results of the analysis of cortical folding in relation to lifestyle risk after correcting the effect of each single lifestyle variable out of the other lifestyle variables using partial correlations. The recurrent associations between higher lifestyle risk and reduced cortical folding in the left dorsal premotor Cortex (PMC) and the right ventro-lateral prefrontal Cortex (PFC) are highlighted in rose and orange, respectively. Results shown in this figure are not adjusted for the combined genetic risk score (GRS). Explanations of abbreviations can be found in Supplementary Figure 1. Abbreviations: n.s. = not significant.

**
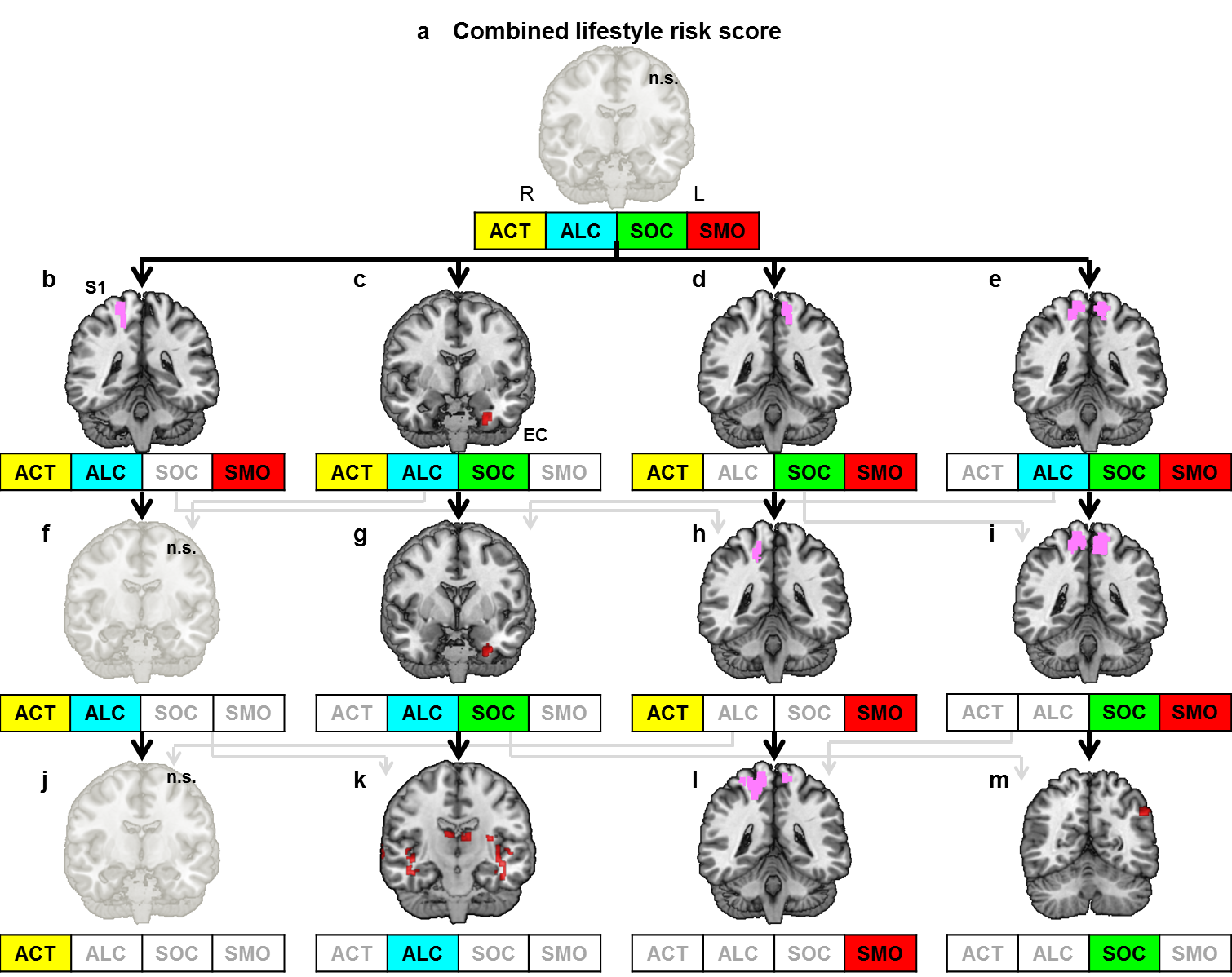
**

**Supplementary Figure 9: Sensitivity analysis for the association between lifestyle risk and RSFC of the seed in dPMC.** Figure represents regions showing increased RSFC to the seed in dPMC in relation to increased lifestyle risk after correcting the influence of each single lifestyle variable out of the other lifestyle variables using partial correlations. Explanations of abbreviations can be found in Supplementary Figure 1.

**
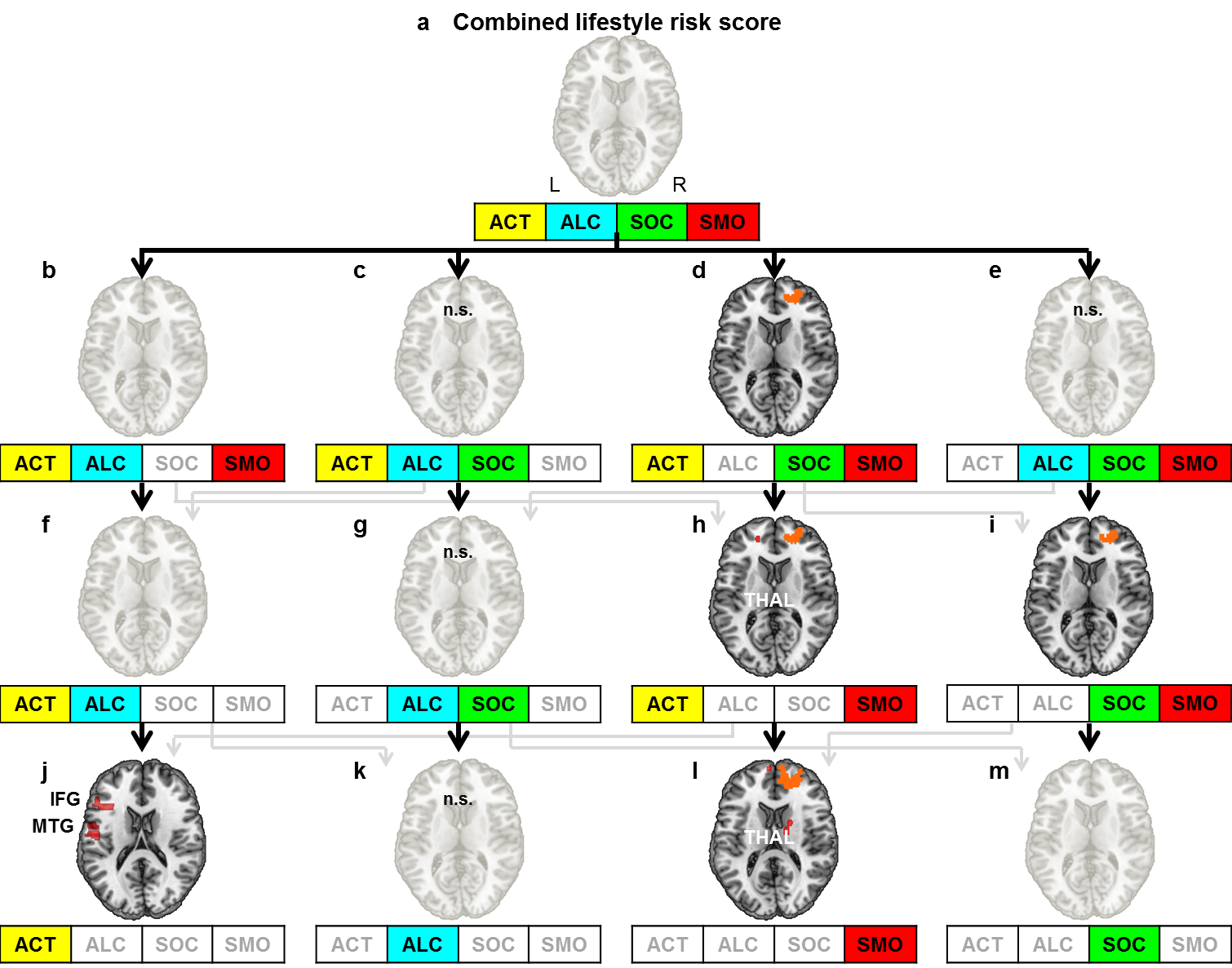
**

**Supplementary Figure 10: Sensitivity analysis for the association between lifestyle risk and RSFC of the seed in vlPFC.** Figure represents GM voxels showing increased RSFC to the seed in vlPFC in relation to increased lifestyle risk after correcting the influence of each single lifestyle variable out of the other lifestyle variables using partial correlations. Explanations of abbreviations can be found in Supplementary Figure 1.


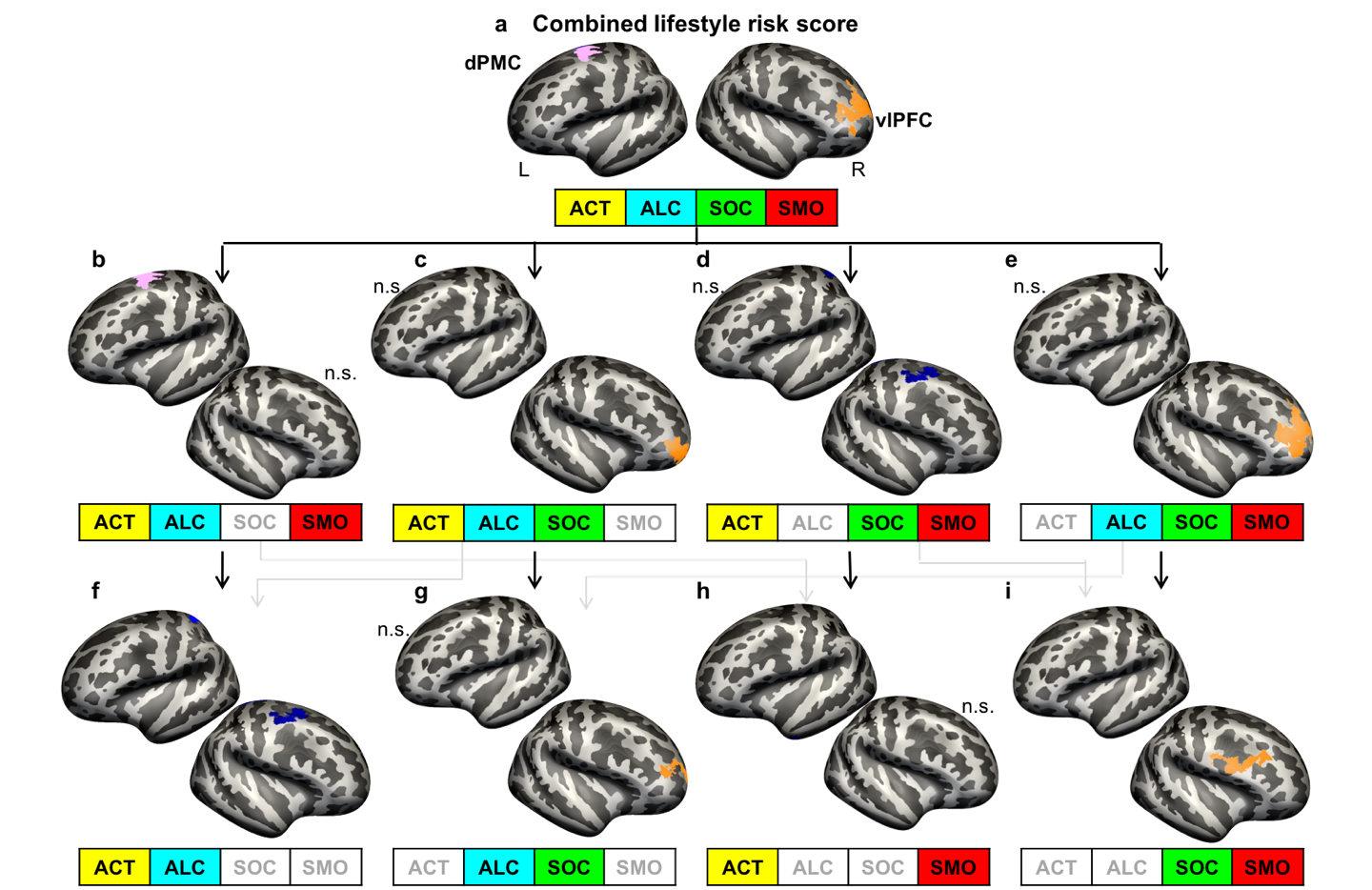


**Supplementary Figure 11:** **Brain regions showing alterations in cortical folding associated with lifestyle risk when additionally adjusting for depressive symptomatology.**

Associations between different risk score models and cortical folding when additionally adjusting for depressive symptomatology as measured with the Beck´s depression inventory score (BDI-II). All results are depicted on the inflated surfaces of fsaverage. The recurrent associations between higher lifestyle risk and reduced cortical folding in the left dorsal premotor Cortex (PMC) and the right ventro-lateral prefrontal Cortex (PFC) are highlighted in pink and orange, respectively. Other negative associations are depicted in blue. n.s. = not significant, dPMC = dorsal PMC, vlPFC = ventro-lateral prefrontal cortex.

**
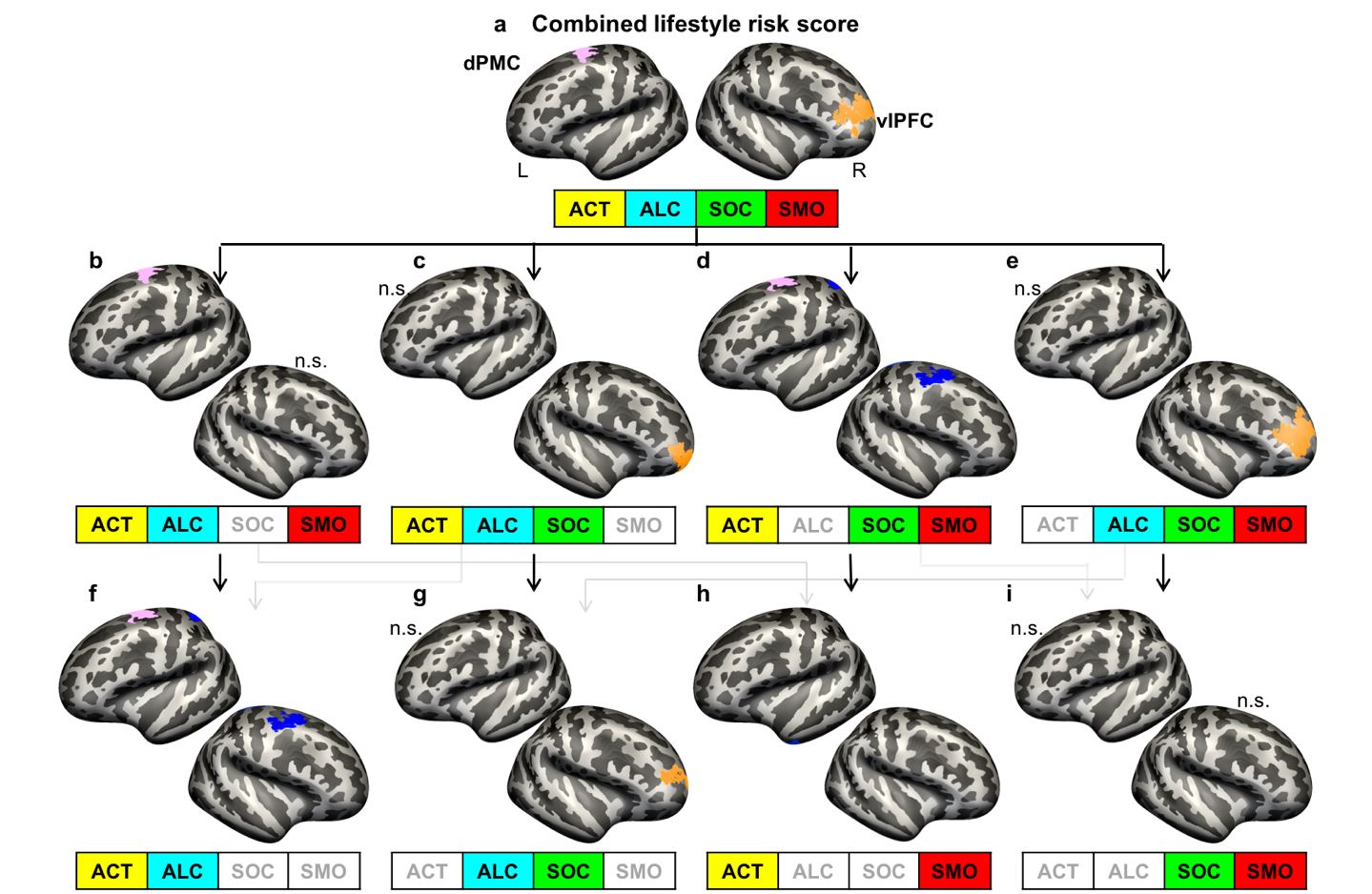
**

**Supplementary Figure 12:** **Brain regions showing alterations in cortical folding associated with lifestyle risk when additionally adjusting for education.**

Associations between different risk score models and cortical folding when additionally adjusting for education as measured with the international standard classification for education (ISCED, Unesco, 2011). All results are depicted on the inflated surfaces of fsaverage. The premotor Cortex (PMC) and the right ventro-lateral prefrontal Cortex (PFC) are highlighted in pink and orange, respectively. Other negative associations are depicted in blue. n.s. = not significant, dPMC = dorsal PMC, vlPFC = ventro-lateral prefrontal cortex.

**
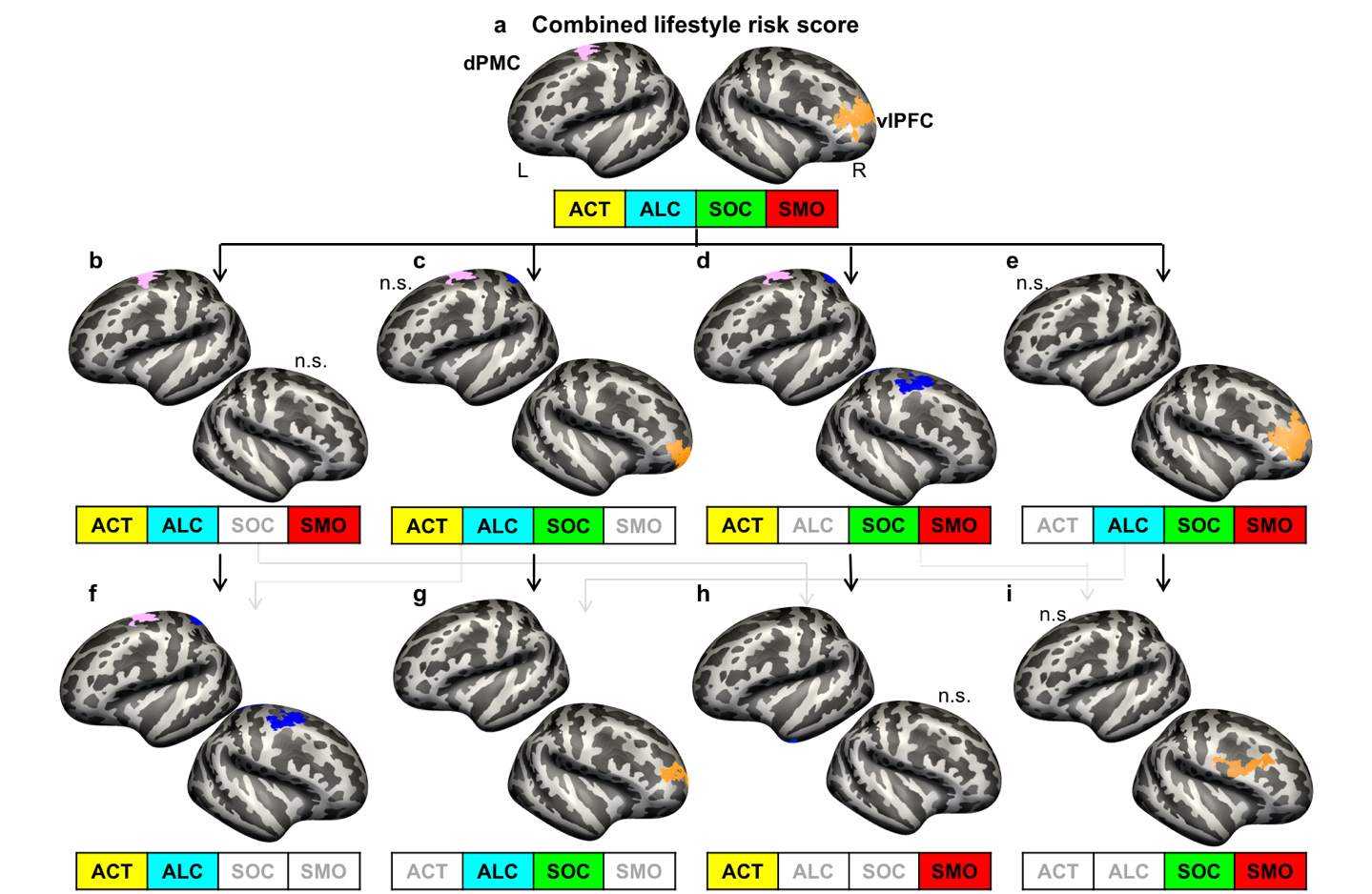
**

**Supplementary Figure 13:** **Brain regions showing alterations in cortical folding associated with lifestyle risk when additionally adjusting for depressive symptomatology and education.**

Associations between different risk score models and cortical folding when additionally adjusting for education as measured with the international standard classification for education (ISCED, Unesco, 2011) and depressive symptomatology as measured with Beck´s depression inventory (BDI-II). All results are depicted on the inflated surfaces of fsaverage. The recurrent associations between higher lifestyle risk and reduced cortical folding in the left dorsal premotor Cortex (PMC) and the right ventro-lateral prefrontal Cortex (PFC) are highlighted in rose and orange, respectively. n.s. = not significant, dPMC = dorsal PMC, vlPFC = ventro-lateral prefrontal cortex.

*
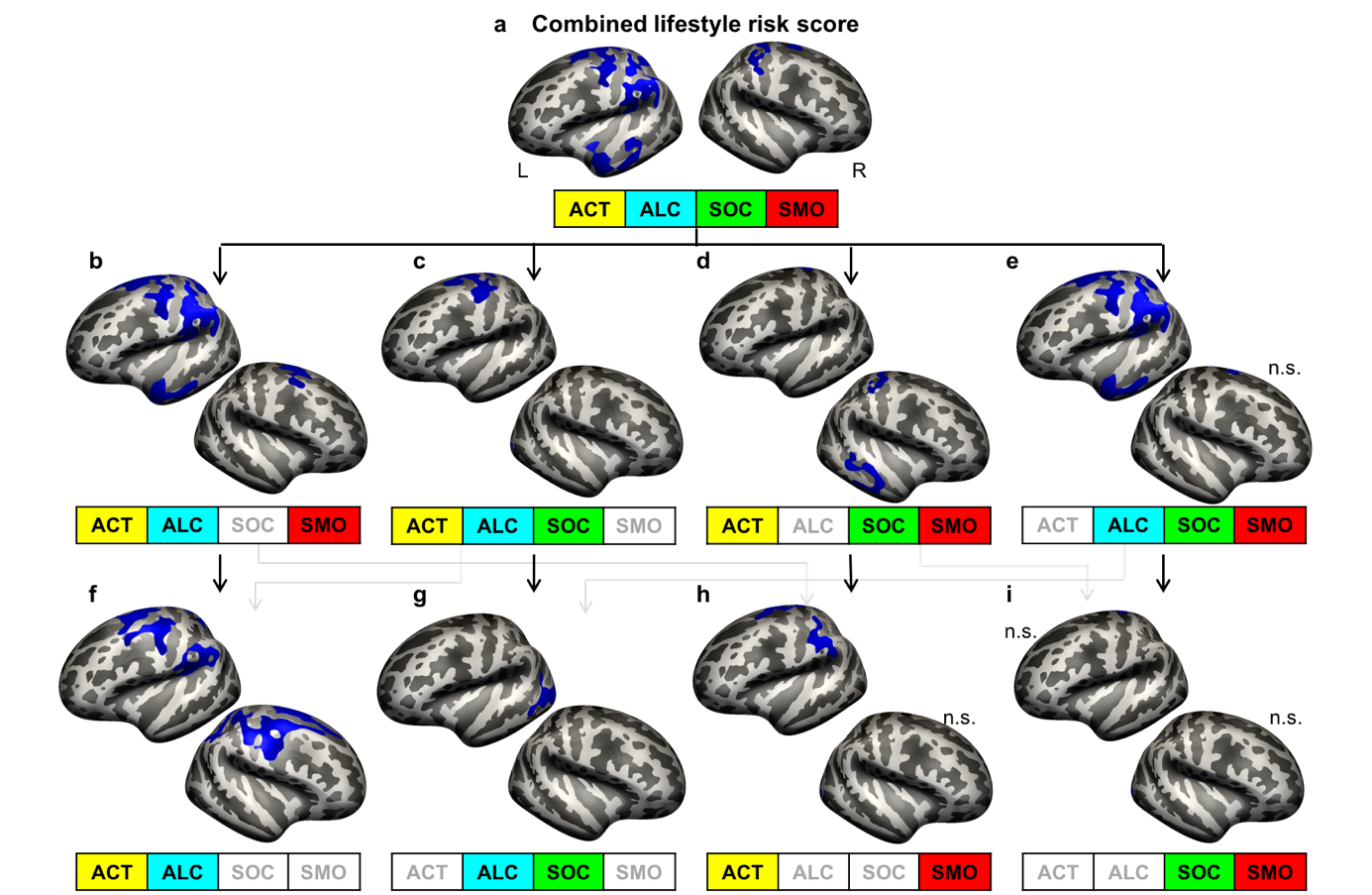
*

**Supplementary Figure 14. Brain regions showing alterations in cortical thickness associated with lifestyle risk in different risk score models (with stepwise exclusion).** All results are corrected for age and gender and corrected for multiple comparisons using Monte Carlo Z simulations with α = 0.05. Positive associations between risk score models and cortical thickness were not found.

**
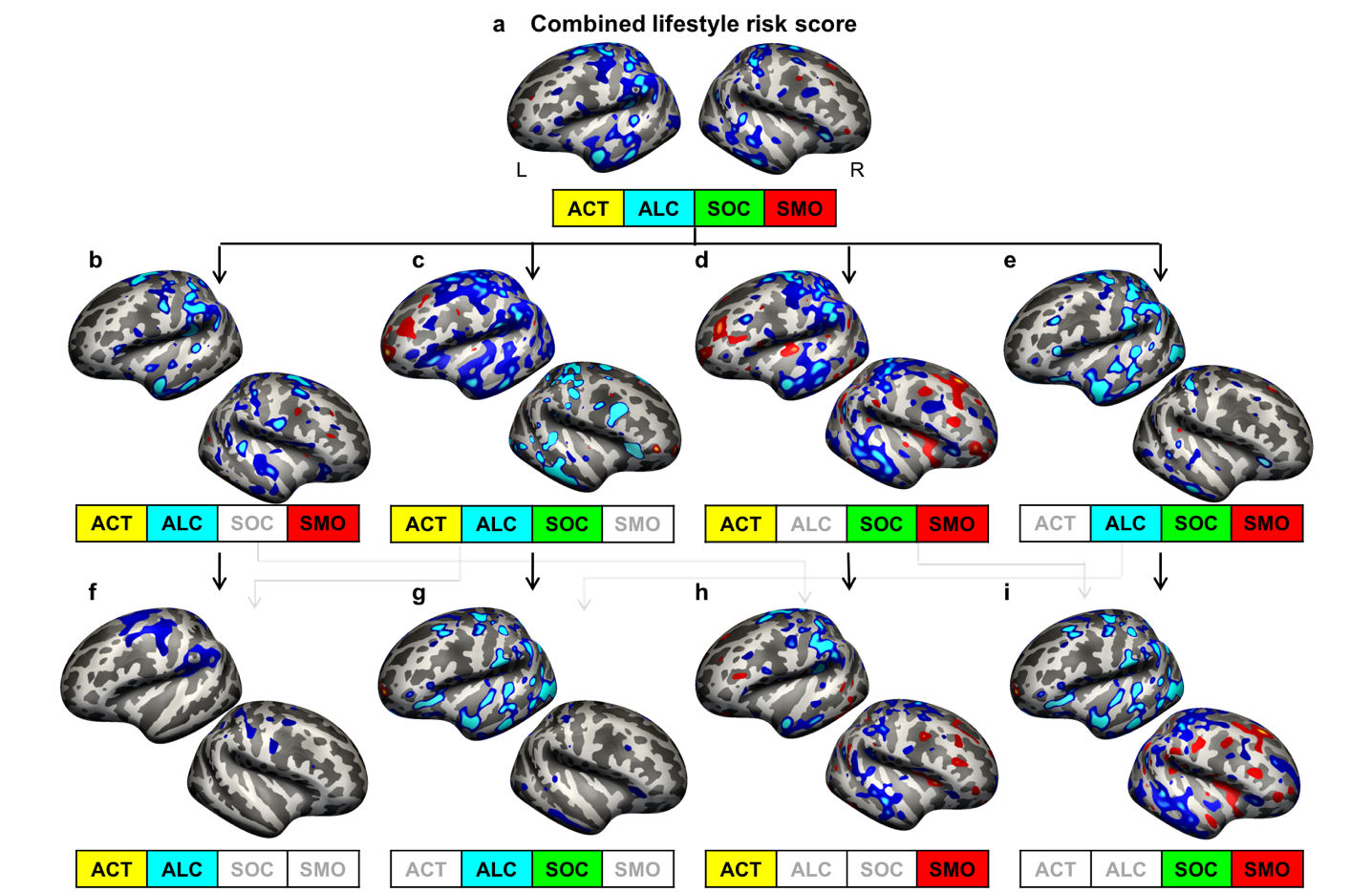
**

**Supplementary Figure 15: Associations between lifestyle risk and differences in cortical thickness without correction for multiple comparisons.**

Associations between different risk score models and cortical thickness without any correction for multiple comparisons. For further conventions and abbreviations please see Suppl. Fig. 1.

**
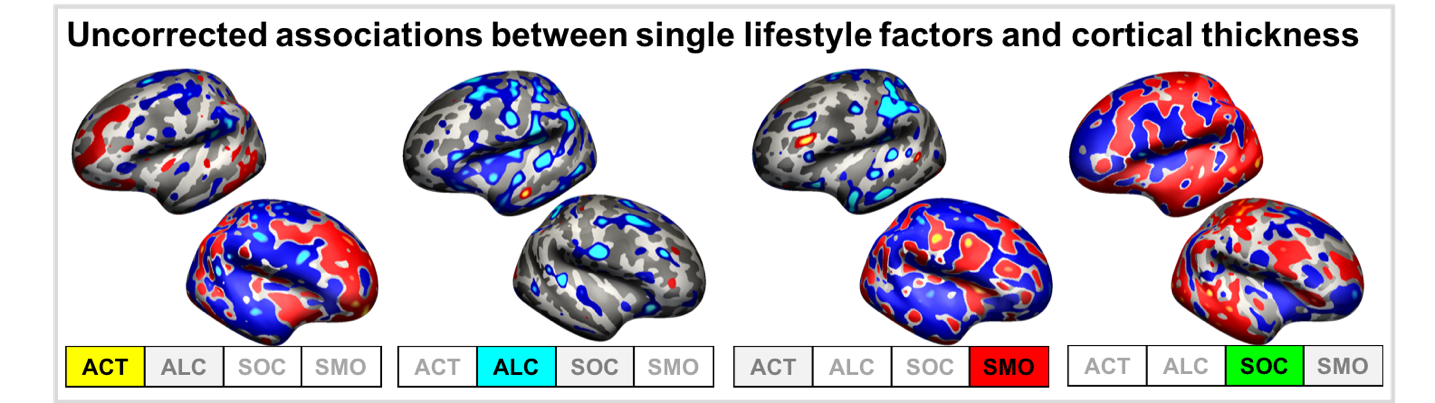
**

**Supplementary Figure 16: Brain regions showing alterations in cortical thickness associated with lifestyle risk without correction for multiple comparisons.**

Associations between single lifestyle variables and cortical thickness without any correction for multiple comparisons depicted on the inflated surfaces of the fsaverage brain. For further conventions and abbreviations please see Suppl. Fig. 1.

**B**

**A**

**Supplementary Figure 17: Beta weights of the multiple linear regression using extracted cortical folding values of the dPMC and vlPFC.** Plots show beta weights of each single lifestyle factor, sex and age as estimated in the first model of the multiple linear regression calculated in SPSS using extracted cortical folding values of the dPMC (A) and vlPFC (B) as dependent variable. Error bars refer to standard errors of the individual beta weights.

**Supplementary Table 1. All possible combinations of lifestyle risk score models.**

| **Risk score model** | **Included lifestyle variables** |
| --- | --- |
| ACT, ALC, SOC, SMO | The combined lifestyle risk score:  Physical activity, alcohol consumption, social integration, smoking |
| ACT, ALC, SMO | Physical activity, alcohol consumption, smoking |
| ACT, ALC, SOC | Physical activity, alcohol consumption, social integration |
| ACT, SOC, SMO | Physical activity, social integration, smoking |
| ALC, SOC, SMO | Alcohol consumption, social integration, smoking |
| ACT, ALC | Physical activity, alcohol consumption |
| ALC, SOC | Alcohol consumption, social integration |
| ACT, SMO | Physical activity, smoking |
| SOC, SMO | Social integration, smoking |

**Supplementary Table 2: Overlap between probability maps of the JuBrain atlas^4-7^ and regions showing significantly decreased cortical folding with higher combined lifestyle risk.**

| **Risk score model** | **Hemis-phere** | **Anatomical landmark** | **Cytoarchitectonically defined region^4-27^** | ***Cwp*-value** |
| --- | --- | --- | --- | --- |
| Combined risk score  **ACT, ALC, SMO, SOC** | left | Premotor cortex (PMC) | - | 0.0001 |
|  | right | Frontal pole extending to inferior frontal gyrus (IFG) | Fp1, Fp2, Area 45 | 0.0001 |
| **ACT, ALC, SMO** | left | PMC | - | 0.0029 |
|  | right | n.s. | - |  |
| **ACT, ALC, SOC** | left | PMC | - | 0.0001 |
|  | right | Frontal pole, middle orbital gyrus | Fp1, Fp2, Fo2 | 0.0001 |
|  |  | IFG | Area 44, 45 | 0.0001 |
|  |  | PMC | - | 0.0001 |
|  |  | Superior parietal lobule (SPL), extending to intra-parietal sulcus (IPS) and superior occipital gyrus | 7A, 7M, 7P, 7PC, hIP3 [IPS], hoc4d (V3A), hoc3d (V3d) | 0.0001 |
| **ACT, SMO, SOC** | left | Frontal pole | Fp1, Fp2 | 0.0007 |
|  |  | PMC | - | 0.0001 |
|  |  | Fusiform gyrus | FG3, FG4 | 0.0079 |
|  | right | Frontal pole, extending to IFG | Fp1, Fp2, Area 44, 45 | 0.0001 |
|  |  | Fusiform gyrus | FG4 | 0.0069 |
| **ALC, SOC, SMO** | left | Frontal pole | Fp1, Fp2 | 0.0090 |
|  |  | PMC | - | 0.0142 |
|  | right | Frontal pole | Fp1, Fp2 | 0.0008 |
|  |  | SFG, MFG | - | 0.0001 |
| **ACT, ALC** | left | PMC | - | 0.0002 |
|  |  | SPL, extending to sensori-motor region | 5A, 5M, 7A, 7PC  Area 4a, Area 2 | 0.0015 |
|  | right | Premotor to sensori-motor cortex | Area 1, 2, 3b, 4a | 0.0001 |
|  |  | SPL | 5L | 0.0001 |
| **ALC, SOC** | left | Frontal Pole | Fp1, Fp2 | 0.0009 |
|  |  | IFG | Area 45 | 0.0042 |
|  |  | PMC | - | 0.0236 |
|  | right | Frontal pole | Fp1, Fp2 | 0.0019 |
|  |  | IFG | Areas 44, 45 | 0.0020 |
|  |  | PMC, M1 | Area 4p | 0.0001 |
|  |  | SPL, extending to IPS and superior occipital gyrus | 7A, 7M, 7P^,^ hIP3 [IPS]^,^ hOC4d (V3A) | 0.0001 |
| **ACT, SMO** | left | PMC | - | 0.0080 |
|  |  | Parietal operculum | OP4 | 0.0093 |
| **SMO, SOC** | left | Frontal pole | Fp1, Fp2 | 0.0001 |
|  | right | Prefrontal cortex | Fp1, Fp2 | 0.0001 |

*Note.* This table summarizes the overlap between the macroanatomical localization of the reported effects with cytoarchitectonical maps of the JuBrain atlas^1-24^. Abbreviations: n.s. = not significant.

**Supplementary Table 3: Overlap between probability maps of the JuBrain atlas with brain regions showing significantly increased RSFC to the seed in left dPMC.**

| **Risk score model** | **Hemi**  **sphere** | **Anatomical landmark** | **Cytoarchitectonically defined region^4-27^** | **Voxel size** |
| --- | --- | --- | --- | --- |
| Combined risk score  **ACT, ALC, SMO, SOC** | left | Entorhinal cortex | - | 72 |
|  |  | S1 / M1 | Areas 3a, 4a | 60 |
|  |  | V3 (dorsal occipital cortex) | Area hOc4d [V3A] | 41 |
|  |  | M1 | Area 4a | 37 |
|  | right | M1 | Area 4a | 83 |
|  |  | M1 | Area 4a | 76 |
| **ACT, ALC, SOC** | left | Enthorinal cortex | - | 33 |
| **ACT, ALC, SMO** | left | V3 (dorsal occipital cortex) | Area hOc4d [V3A] | 71 |
|  | right | M1 | Area 4a | 110 |
|  |  | S1 / M1 | Areas 3b, 4a | 107 |
|  |  | Middle temporal gyrus | - | 45 |
| **ACT, SMO, SOC** | left | M1 | - | 114 |
|  |  | S1 | Area 3a | 69 |
|  |  | V3 (dorsal occipital cortex) | Area hOc4d [V3A] | 51 |
|  |  | Medial frontal gyrus | - | 49 |
|  | right | M1 | Area 4a | 87 |
|  |  | M1 / S1 | Areas 4a, 3b | 79 |
|  |  | M1 (precentral gyrus) | - | 45 |
|  |  | Medial frontal gyrus | - | 41 |
| **ALC, SMO, SOC** | left | paracentral region | - | 128 |
|  |  | M1 (precentral gyrus) | Area 4a | 99 |
|  |  | Entorhinal cortex | Entorhinal cortex, Subiculum | 89 |
|  |  | S1, extending to SPL | Area 3a, 5M (SPL) | 74 |
|  | right | Paracentral region | 4a, 3b | 467 |
|  |  | Entorhinal cortex | entorhinal cortex | 62 |
| **ACT, SMO** | left | V3 (dorsal occipital cortex) | Area hOc4d [V3A] | 106 |
|  |  | M1 | Area 4p | 50 |
|  | right | M1 | Area 4a | 159 |
|  |  | S1 / M1 | Areas 3b, 4a | 154 |
| **SOC** (as a single variable) | left | Temporo-parietal junction | Areas PGa (IPL), PFm (IPL) | 59 |
| **SMO** (as a single variable) | left | M1 | Area 4a | 255 |
|  |  | S1 | Area 3a | 64 |
|  |  | V3 (dorsal occipital cortex) | Area hOc4d [V3A] | 53 |
|  |  | Superior frontal gyrus |  | 37 |
|  | right | M1 | Area 4a | 945 |
|  |  | M1 | - | 54 |
|  |  | S1 | Area 2 | 37 |
|  |  | V3 (dorsal occipital cortex) | Area hOc4la [V3A] | 32 |
| **ALC** (as a single variable) | left | Cerebellum | - | 118 |
|  |  | Brainstem | - | 57 |
|  | right | Cerebellum | - | 56 |
|  |  | Hippocampus | - | 35 |

*Note.* Abbreviations: n.s. = not significant.

**Supplementary Table 4: Overlap between probability maps of the JuBrain atlas with brain regions showing significantly increased RSFC to the seed in right vlPFC.**

| **Risk score model** | **Hemi**  **sphere** | **Anatomical landmark** | **Cytoarchitectonically defined region^4-27^** | **Voxel size** |
| --- | --- | --- | --- | --- |
| Combined risk score  **ACT, ALC, SMO, SOC** | right | Superior frontal gyrus | Fp1 | 64 |
| **ACT, SMO, SOC** | right | Superior frontal gyrus | Fp1 | 58 |
|  |  | Superior medial gyrus | Fp2 | 51 |
| **ACT, ALC, SMO** | right | Super frontal gyrus | Fp1 | 69 |
|  |  | Anterior cingulum | - | 35 |
| **ACT, ALC, SOC** | right | Orbitofrontal gyrus | - |  |
| **ALC, SOC, SMO** | right | Superior frontal gyrus | Fp1 | 171 |
|  |  | Temporo-parietal junction | Areas PGa (IPL), PFm (IPL) | 34 |
| **ACT, ALC** | left | Cerebellum |  |  |
| **ACT, SMO** | left | Superior frontal gyrus | Fp1 | 80 |
| **ACT, SMO** | right  bilateral | Superior frontal gyrus | Fp1 | 274 |
|  |  | Thalamus | - | 61 |
|  | right | Middle frontal gyrus | - | 33 |
| **SMO, SOC** | right | Superior frontal gyrus | Fp1 | 117 |
| **SMO** (as a single variable) | left | Middle frontal gyrus | - | 94 |
|  |  | Superior frontal gyrus | - | 83 |
|  | right | Superior frontal gyrus | Fp1 | 389 |
|  | bilateral | Thalamus | - | 60 |
| **SOC** (as a single variable) | right | Caudate nucleus | - | 112 |
|  |  | Inferior frontal gyrus,  p. orbitalis | - | 53 |
| **ACT** (as a single variable) | left | Inferior frontal gyrus,  p. triangularis | Area 45 | 109 |
|  |  | Postcentral gyrus | - | 93 |
|  | right | Middle temporal gyrus | hOc4la | 88 |

*Note.* Abbreviations: n.s. = not significant.

**Supplementary Table 5: Overlap between probability maps of the JuBrain atlas with brain regions showing significantly decreased RSFC to the seed in left PMC.**

| **Risk score model** | **Hemi**  **sphere** | **Anatomical landmark** | **Cytoarchitectonically defined region^4-27^** | **Voxel size** |
| --- | --- | --- | --- | --- |
| Combined risk score  **ACT, ALC, SMO, SOC** | right | Precentral sulcus | - | 45 |
| **SMO** (as a single variable) | left | Cerebellum | - | 33 |
| **SOC** (as a single variable) | left | M1 | Area 4a | 42 |
| **ACT** (as a single variable) | left | Paracingulate sulcus | - | 37 |

**Supplementary Table 6: Overlap between probability maps of the JuBrain atlas with brain regions showing significantly decreased RSFC to the seed in right vlPFC.**

| **Risk score model** | **Hemi**  **sphere** | **Anatomical landmark** | **Cytoarchitectonically defined region^4-27^** | **Voxel size** |
| --- | --- | --- | --- | --- |
| **ACT, ALC, SMO** | right | Dorsal occipital cortex | - | 43 |
| **ACT, ALC** | right | Dorsal occipital cortex | - | 70 |
| **ALC** (as a single variable) | left | Inferior frontal gyrus | Area 44 | 53 |
| **ACT** (as a single variable) | right | Cerebellum | - | 40 |

**Supplementary Table 7: Regions showing significantly decreased cortical folding with higher integrated lifestyle risk when additionally adjusting for polygenic risk.**

| **Risk score model** | **Hemis-phere** | **Anatomical landmark** | **Cytoarchitectonically defined region^4-27^** | ***Cwp*-value** |
| --- | --- | --- | --- | --- |
| Combined risk score  **ACT, ALC, SMO, SOC** | left | Premotor cortex (PMC) | - | 0.00010 |
|  | right | Frontal pole, extending to  inferior frontal gyrus (IFG) | Fp1, Fp2, Area 45 | 0.00010 |
|  |  | Precuneus | 7A, 7P | 0.00010 |
| **ACT, ALC, SMO** | left | n.s. | - | - |
|  | right | n.s. | - | - |
| **ACT, ALC, SOC** | left | PMC | - | 0.00010 |
|  | right | Frontal pole, middle orbital gyrus | Fp1, Fp2, Fo2 | 0.00010 |
|  |  | IFG | Area 44, 45 | 0.00010 |
|  |  | Precentral gyrus | 4a, 4p | 0.00010 |
|  |  | Precuneus | 7A, 7P, hOc4d [V3A] | 0.00010 |
| **ACT, SMO, SOC** | left | PMC | - | 0.00070 |
|  |  | Frontal Pole | Fp1, Fp2 | 0.00010 |
|  |  | Fusiform gyrus | FG3, FG4 | 0.00010 |
|  | right | Frontal pole, extending to IFG | Fp1, Fp2, Area 45 | 0.00010 |
|  |  | Precuneus, extending to fusiform gyrus | FG3, FG4 | 0.00010 |
| **ALC, SOC, SMO** | left | Frontal pole | Fp1, Fp2 | 0.00010 |
|  |  | PMC | - | 0.00010 |
|  |  | Fusiform gyrus | FG3, FG4 | 0.00010 |
|  | right | Frontal pole, extending to IFG | Fp1, Fp2, Area 44 | 0.00010 |
|  |  | SPL, extending to intra-parietal sulcus | 7A, 7M (SPL), hIP3 (IPS] | 0.00010 |
|  |  | Precuneus, extending to calcarine sulcus and fusiform gyrus | hOc1 [V1], hOc2 [V2], FG3, FG4 | 0.00010 |
| **ACT, ALC** | left | SPL | 5L, 7A | 0.00480 |
|  | right | Precentral gyrus | 4a | 0.00287 |
| **ALC, SOC** | left | Frontal Pole | Fp1, Fp2 | 0.00200 |
|  |  | IFG | Area 45 | 0.03190 |
|  |  | PMC | - | 0.00010 |
|  | right | Frontal pole, extending to IFG | Fp1, Fp2, Areas 44, 45 | 0.00010 |
|  |  | PMC | - | 0.00010 |
|  |  | Superior temporal sulcus | - | 0.00180 |
| **ACT, SMO** | left | n.s. | - | - |
|  | right | Fusiform gyrus, extending to lateral occipital cortex | FG3, FG4, hOc4v [V4 (v)] | 0.00010 |
| **SMO, SOC** | left | Frontal pole | Fp1, Fp2 | 0.00010 |
|  |  | Premotor cortex | - | 0.00100 |
|  |  | Fusiform gyrus | FG3, FG4 | 0.00010 |
|  | right | Frontal Pole, extending to superior frontal gyrus | Fp1, Fp2 | 0.00010 |
|  |  | Superior parietal lobule, extending to intra-parietal sulcus | 7A [SPL], hIP3 [SPL] | 0.00010 |

*Note.* Abbreviations: n.s. = not significant.

**Supplementary Table 8: Overlap between probability maps of the JuBrain atlas with brain regions showing significantly increased RSFC to the seed in left dPMC when additionally adjusting for polygenic risk.**

| **Risk score model** | **Hemi**  **sphere** | **Anatomical landmark** | **Cytoarchitectonically defined region^4-27^** | **Voxel size** |
| --- | --- | --- | --- | --- |
| Combined risk score  **ACT, ALC, SMO, SOC** | left | Entorhinal cortex | Entorhinal cortex, Subiculum | 76 |
|  |  | S1 / M1 | Areas 3a, 4a, 5L (SPL) | 64 |
|  |  | M1 | Area 4a | 46 |
|  | right | M1 | Area 4a | 79 |
|  |  | M1 | Area 4a, 3b, 5L (SPL) | 72 |
|  | bilateral | Brainstem | - | 33 |
| **ACT, ALC, SOC** | left | Entorhinal cortex | Entorhinal cortex | 47 |
| **ACT, ALC, SMO** | left | V3 (superior occipital gyrus) | Area hOc4d [V3A] | 50 |
|  | right | M1 | Area 4a, 3b | 133 |
|  |  | M1 | Area 4a | 125 |
|  |  | Middle temporal gyrus | - | 36 |
| **ACT, SMO, SOC** | left | M1 | 4a | 99 |
|  |  | S1, extending to SPL | 3a, 5M | 84 |
|  |  | Superior occipital gyrus | hOc4d [V3A] | 43 |
|  |  | Medial frontal gyrus | - | 42 |
|  |  | Medial frontal gyrus | - | 38 |
|  | right | M1 / S1 | Area 4a, 3b | 73 |
|  |  | M1 | Area 4a | 65 |
| **ALC, SMO, SOC** | left | M1 | Area 4a | 121 |
|  |  | M1 / S1, extending to SPL | 4a, 3a, 5M | 75 |
|  |  | Medial frontal gyrus | - | 74 |
|  |  | Entorhinal cortex | Entorhinal cortex | 71 |
|  |  | Paracentral cortex | Entorhinal cortex | 47 |
|  | right | M1 | Area 4a | 416 |
| **ACT, SMO** | right | M1 | Area 4a | 153 |
|  |  | M1 | Area 4a, 3b | 145 |
|  | left | V3 (superior occipital gyrus) | Area hOc4d [V3A] | 106 |
|  |  | M1 | 4p, 4a | 53 |
| **SOC, SMO** | left | M1 | Area 4a | 192 |
|  |  | Paracentral cortex | Areas 4a, 3a, 5M | 128 |
|  |  | Medial frontal gyrus | - | 101 |
|  | right | Paracentral cortex | Area 4a, 3b, 5M | 549 |
|  |  | Medial frontal gyrus | - | 41 |
|  |  | M1 | - | 38 |
|  |  | SMA | - | 37 |
| **SOC** (as a single variable) | left | Temporo-parietal junction | Areas PGa (IPL), PFm (IPL) | 56 |
| **SMO** (as a single variable) | left | M1 | Area 4a | 229 |
|  |  | S1 | Area 3a | 54 |
|  |  | V3 (superior occipital gyrus) | Area hOc4d [V3A] | 51 |
|  |  | M1 | Area 4a | 49 |
|  | right | M1 | Area 4a, 3b, 5L | 967 |
|  |  | Medial frontal gyrus | - | 46 |
|  |  | S1 | Area 2 | 42 |
|  |  | Superior frontal gyrus | - | 35 |
|  |  | Posterior middle temporal gyrus | Area hOc4la | 31 |

**Supplementary Table 9: Overlap between probability maps of the JuBrain atlas with brain regions showing significantly increased RSFC to the seed in right vlPFC when additionally adjusting for polygenic risk.**

| **Risk score model** | **Hemi**  **sphere** | | **Anatomical landmark** | **Cytoarchitectonically defined region^4-27^** | | **Voxel size** |
| --- | --- | --- | --- | --- | --- | --- |
| Combined risk score  **ACT, ALC, SMO, SOC** | right | | Superior frontal gyrus | Fp1 | 68 | |
| **ACT, ALC, SMO** | right | | Superior frontal gyrus | Fp1 | 65 | |
| **ACT, SMO, SOC** | right | | Superior frontal gyrus | Fp1 | 171 | |
|  |  |  | Supramaginal gyrus | - | 34 | |
| **ACT, SMO** | left | | Superior frontal gyrus | Fp1 | 54 | |
|  | right | | Superior frontal gyrus | Fp1 | 259 | |
|  |  |  | Middle frontal gyrus | Fp1 | 37 | |
| **SMO, SOC** | right | | Superior frontal gyrus | Fp1 | 59 | |
|  |  |  | Superior medial gyrus | - | 50 | |
|  |  |  | Insula | - | 37 | |
| **SMO** (as a single variable) | left | | Superior frontal gyrus | - | 81 | |
|  |  |  | Middle frontal gyrus | - | 85 | |
|  | right | | Superior frontal gyrus | Fp1 | 311 | |
| **SOC** (as a single variable) | right | | Caudate nucleus | - | 53 | |
|  |  |  | Inferior frontal gyrus,  orbital part | - | 51 | |
| **ACT** (as a single variable) | left |  | Inferior frontal gyrus,  triangular part | Area 45 | 76 | |
|  |  |  | Postcentral Gyrus | OP4 | 52 | |
|  | right |  | V3 (dorsal occipital cortex) | Area hOc4la | 66 | |

**Supplementary Table 10: Regions showing significantly decreased cortical folding with higher combined lifestyle risk in the sensitivity analysis.**

| **Risk score model** | **Hemis-phere** | **Anatomical landmark** | **Cytoarchitectonically defined region^4-27^** | **Cwp-value** |
| --- | --- | --- | --- | --- |
| Combined risk score  **ACT, ALC, SMO, SOC** | left | Premotor cortex (PMC) | - | 0.00010 |
|  | right | Frontal pole, extending to Inferior frontal gyrus (IFG) | Fp1, Fp2, Areas 45 | 0.00010 |
|  |  | PMC | - | 0.00020 |
| **ACT, ALC, SMO** | left | PMC | - | 0.00020 |
|  | right | n.s. | - | - |
| **ACT, ALC, SOC** | left | PMC | - | 0.00010 |
|  | right | Frontal pole, middle orbital gyrus | Fp1, Fp2, Fo2 | 0.00010 |
|  |  | IFG | Area 44, 45 | 0.00010 |
|  |  | PMC, extending to M1 | 4a, 4p | 0.00010 |
|  |  | SPL, extending to cuneus | 7A, 7P, hOc4d [V3A] | 0.00010 |
| **ACT, SMO, SOC** | left | Frontal pole | Fp1, Fp2 | 0.00020 |
|  |  | PMC | - | 0.00010 |
|  | right | Frontal pole, extending to IFG | Fp1, Fp2, Area 44 | 0.00010 |
|  |  | PMC | - | 0.00010 |
|  | right | Frontal pole | Fp1, Fp2 | 0.00010 |
|  |  | PMC | - | 0.00170 |
| **ACT, ALC** | left | PMC | - |  |
|  |  | SPL | 5L | 0.00140 |
|  | right | Premotor cortex | - | 0.00010 |
|  |  | Sensory-motor cortex, extending to SPL | Area 4, 2, 5L (SPL) | 0.00010 |
| **ALC, SOC** | left | Frontal Pole | Fp1, Fp2 | 0.00390 |
|  |  | IFG | Area 45 | 0.00730 |
|  | right | Frontal pole, extending to IFG | Fp1, Fp2, Areas 44, 45 | 0.00010 |
|  |  | PMC, extending to inferior M1 | 3b | 0.00590 |
|  |  | Superior M1 | 4a | 0.00180 |
|  |  | SPL, Precuneus | 7A, 7P (SPL) | 0.00010 |
|  |  | Middle temporal gyrus | - | 0.00210 |
| **ACT, SMO** | left | PMC | - | 0.00680 |
|  | right | n.s. | - |  |
| **SMO, SOC** | left | Frontal pole | Fp1, Fp2 | 0.00010 |
|  | right | Frontal Pole, extending to middle frontal gyrus | Fp1, Fp2 | 0.00010 |

**Supplementary Table 11: Overlap between probability maps of the JuBrain atlas with brain regions showing significantly increased RSFC to the seed in left dPMC in the sensitivity analyses.**

| **Risk score model** | **hemi**  **sphere** | **anatomical landmark** | **cytoarchitectonically defined region^4-27^** | **voxel size** |
| --- | --- | --- | --- | --- |
| Combined risk score  **ACT, ALC, SMO, SOC** | left | Entorhinal cortex | Entorhinal cortex | 67 |
| **ACT, ALC, SMO** | right | S1 / M1 | Areas 3b , 4a | 52 |
|  |  | M1 | 4a | 37 |
| **ACT, ALC, SOC** | left | Entorhinal cortex | Entorhinal cortex | 37 |
| **ACT, SMO, SOC** | left | M1 | - | 114 |
|  |  | S1 | Area 3a | 69 |
|  |  | V3 (superior occipital gyrus) | Area hOc4d [V3A] | 51 |
|  |  | Medial frontal gyrus | - | 49 |
|  | right | M1 | Area 4a | 87 |
|  |  | M1 / S1 | Areas 4a, 3b | 79 |
|  |  | M1 (precentral gyrus) | - | 45 |
|  |  | Medial frontal gyrus | - | 41 |
| **ALC, SMO, SOC** | left | Paracentral lobule | - | 128 |
|  |  | M1 (precentral gyrus) | Area 4a | 99 |
|  |  | Entorhinal cortex | - | 89 |
|  |  | SPL (precuneus) | Area 5M (SPL) | 74 |
|  | right | M1 | Area 4a | 467 |
|  |  | Entorhinal cortex | - | 62 |
| **ACT, SMO** | left | V3 (dorsal occipital cortex) | Area hOc4d [V3A] | 77 |
|  |  | M1 | Area 4p | 32 |
|  | right | M1 | Area 4a | 80 |
|  |  | S1 / M1 | Areas 3b, 4a | 92 |
| **SOC** (as a single variable) | left | Temporo-parietal junction | Areas PGa (IPL), PFm (IPL) | 59 |
| **SMO** (as a single variable) | left | M1 | Area 4a | 255 |
|  |  | S1 | Area 3a | 64 |
|  |  | V3 (superior occipital gyrus) | Area hOc4d [V3A] | 53 |
|  |  | Superior frontal gyrus |  | 37 |
|  | right | M1 | Area 4a | 945 |
|  |  | M1 | - | 54 |
|  |  | S1 | Area 2 | 37 |
|  |  | V3 (dorsal occipital cortex) | Area hOc4la | 32 |

**Supplementary Table 12: Exact anatomical localization of brain regions showing significantly increased RSFC to the seed in VLPFC in the sensitivity analyses.**

| **Risk score model** | **Hemi**  **sphere** | **Anatomical landmark** | **Cytoarchitectonically defined region^4-27^** | **Voxel size** |
| --- | --- | --- | --- | --- |
| Combined risk score  **ACT, ALC, SMO, SOC** | right | Superior frontal gyrus | Fp1 | 64 |
| **ACT, SMO, SOC** | right | Superior frontal gyrus | Fp1 | 126 |
|  |  | Superior medial gyrus | Fp2 | 42 |
| **ACT, SMO** | left | Superior frontal gyrus | Fp1 | 80 |
| **ACT, SMO** | left | Superior frontal gyrus | - | 62 |
|  | right | Superior frontal gyrus | Fp1 | 237 |
|  |  | Middle frontal gyrus | - | 45 |
| **SMO, SOC** | right | Superior frontal gyrus | Fp1 | 117 |
| **SMO** (as a single variable) | left | Middle frontal gyrus | - | 94 |
|  |  | Superior frontal gyrus | - | 83 |
|  | right | Superior frontal gyrus | Fp1 | 389 |
|  | bilateral | Thalamus | - | 60 |
| **SOC** (as a single variable) | right | Caudate nucleus | - | 112 |
|  |  | Inferior frontal gyrus,  p. orbitalis | - | 53 |
| **ACT** (as a single variable) | left | Inferior frontal gyrus,  p. triangularis | Area 45 | 109 |
|  |  | Postcentral gyrus | - | 93 |
|  | right | Middle temporal gyrus | hOc4la | 88 |

**References**

1 Hautzinger, M., Keller, F & Kühner, C. Beck Depressions-Inventar, 2nd Edn. (BDI-II). Frankfurt: Harcourt Test Services (2006).

1. Unesco, E. (1997). International Standard Classification of Education-ISCED 1997: November 1997. Unesco.
2. Fischl, B., & Dale, A. M. Measuring the thickness of the human cerebral cortex from magnetic resonance images. *PNAS* **97**, 11050-11055 (2000).
3. Zilles, K., Amunts, K. Centenary of Brodmann’s map – conception and fate. *Nat. Neurosci.* 11, 139-145

5 Eickhoff, S. B., Heim, S., Zilles, K. & Amunts, K. Testing anatomically specified hypotheses in functional imaging using cytoarchitectonic maps. *Neuroimage* **32**, 570-582, doi:10.1016/j.neuroimage.2006.04.204 (2006).

6 Eickhoff, S. B. *et al.* Assignment of functional activations to probabilistic cytoarchitectonic Areas revisited. *Neuroimage* **36**, 511-521 (2007).

7 Eickhoff, S. B. *et al.* A new SPM toolbox for combining probabilistic cytoarchitectonic maps and functional imaging data. *Neuroimage* **25**, 1325-1335, doi:10.1016/j.neuroimage.2004.12.034 (2005).

8 Bludau, S. *et al.* Cytoarchitecture, probability maps and functions of the human frontal pole. *Neuroimage* **93**, 260-275, doi:10.1016/j.neuroimage.2013.05.052 (2014).

9 Amunts, K. *et al.* Broca's region revisited: cytoarchitecture and intersubject variability. *Journal of Comparative Neurology* **412**, 319-341 (1999).

10 Henssen, A. *et al.* Cytoarchitecture and probability maps of the human medial orbitofrontal cortex. *Cortex; a journal devoted to the study of the nervous system and behavior* **75**, 87-112, doi:10.1016/j.cortex.2015.11.006 (2016).

11 Scheperjans, F. *et al.* Probabilistic maps, morphometry, and variability of cytoarchitectonic Areas in the human superior parietal cortex. *Cereb. Cortex* **18**, 2141-2157, doi:10.1093/cercor/bhm241 (2008).

12 Scheperjans, F. *et al.* Observer-independent cytoarchitectonic mapping of the human superior parietal cortex. *Cereb. Cortex* **18**, 846-867, doi:10.1093/cercor/bhm116 (2008).

13 Choi, H. J. *et al.* Cytoarchitectonic identification and probabilistic mapping of two distinct Areas within the anterior ventral bank of the human intraparietal sulcus. *The Journal of comparative neurology* **495**, 53-69, doi:10.1002/cne.20849 (2006).

14 Rottschy, C. *et al.* Ventral visual cortex in humans: cytoarchitectonic mapping of two extrastriate Areas. *Hum. Brain. Mapp.* **28**, 1045-1059, doi:10.1002/hbm.20348 (2007).

15 Lorenz, S. *et al.* Two New Cytoarchitectonic Areas on the Human Mid-Fusiform Gyrus. *Cereb. Cortex,* **27(1),** 373-385, doi:10.1093/cercor/bhv225 (2015).

16 Caspers, J. *et al.* Cytoarchitectonical analysis and probabilistic mapping of two extrastriate Areas of the human posterior fusiform gyrus. *Brain Struct. Funct.* **218**, 511-526, doi:10.1007/s00429-012-0411-8 (2013).

17 Geyer, S. *et al.* Two different Areas within the primary motor cortex of man. *Nature* **382**, 805 (1996).

18 Grefkes, C., Geyer, S., Schormann, T., Roland, P. & Zilles, K. Human somatosensory Area 2: observer-independent cytoarchitectonic mapping, interindividual variability, and population map. *Neuroimage* **14**, 617-631, doi:10.1006/nimg.2001.0858 (2001).

19 Geyer, S., Schleicher, A. & Zilles, K. Areas 3a, 3b, and 1 of human primary somatosensory cortex: 1. Microstructural organization and interindividual variability. *Neuroimage* **10**, 63-83 (1999).

20 Geyer, S., Schormann, T., Mohlberg, H. & Zilles, K. Areas 3a, 3b, and 1 of human primary somatosensory cortex. Part 2. Spatial normalization to standard anatomical space. *Neuroimage* **11**, 684-696, doi:10.1006/nimg.2000.0548 (2000).

21 Eickhoff, S. B., Schleicher, A., Zilles, K. & Amunts, K. The human parietal operculum. I. Cytoarchitectonic mapping of subdivisions. *Cereb. Cortex* **16**, 254-267, doi:10.1093/cercor/bhi105 (2006).

22 Eickhoff, S. B., Amunts, K., Mohlberg, H. & Zilles, K. The human parietal operculum. II. Stereotaxic maps and correlation with functional imaging results. *Cereb. Cortex* **16**, 268-279, doi:10.1093/cercor/bhi106 (2006).

23 Amunts, K. *et al.* Cytoarchitectonic mapping of the human amygdala, hippocampal region and entorhinal cortex: intersubject variability and probability maps. *Anatomy and embryology* **210**, 343-352, doi:10.1007/s00429-005-0025-5 (2005).

24 Caspers, S. *et al.* The human inferior parietal lobule in stereotaxic space. *Brain Struct. Funct.* **212**, 481-495, doi:10.1007/s00429-008-0195-z (2008).

25 Caspers, S. *et al.* The human inferior parietal cortex: cytoarchitectonic parcellation and interindividual variability. *Neuroimage* **33**, 430-448, doi:10.1016/j.neuroimage.2006.06.054 (2006).

26 Malikovic, A. *et al.* Cytoarchitecture of the human lateral occipital cortex: mapping of two extrastriate Areas hOc4la and hOc4lp. *Brain Struct. Funct.* **221**, 1877-1897, doi:10.1007/s00429-015-1009-8 (2016).

27 Amunts, K., Malikovic, A., Mohlberg, H., Schormann, T. & Zilles, K. Brodmann's Areas 17 and 18 brought into stereotaxic space—where and how variable? *Neuroimage* **11**, 66-84 (2000).
