# Supplementary material for: Combining lifestyle risks to disentangle brain structure and functional connectivity differences in older adults
Source: Nat Commun. 2019 Feb 6;10:621. doi: 10.1038/s41467-019-08500-x (PMC6365564; doi:10.1038/s41467-019-08500-x)
Supplement: Supplementary file 3 — Description of Additional Supplementary Files [file 41467_2019_8500_MOESM3_ESM.docx]

**Description of Additional Supplementary Files**

**Supplementary Data 1 -** Full correlation matrix of lifestyle and subcortical structures

The table shows the complete matrix for all spearman correlations between lifestyle risk score models, single lifestyle variables and the examined subcortical structures, corrected for age and gender.
